# PALSSE: A program to delineate linear secondary structural elements from protein structures

**DOI:** 10.1186/1471-2105-6-202

**Published:** 2005-08-11

**Authors:** Indraneel Majumdar, S Sri Krishna, Nick V Grishin

**Affiliations:** 1 Howard Hughes Medical Institute, University of Texas Southwestern Medical Center, 5323 Harry Hines Blvd. Dallas, TX 75390, USA; 2 Department of Biochemistry, University of Texas Southwestern Medical Center, 5323 Harry Hines Blvd., Dallas, TX 75390, USA

## Abstract

**Background:**

The majority of residues in protein structures are involved in the formation of α-helices and β-strands. These distinctive secondary structure patterns can be used to represent a protein for visual inspection and in vector-based protein structure comparison. Success of such structural comparison methods depends crucially on the accurate identification and delineation of secondary structure elements.

**Results:**

We have developed a method **PALSSE (Predictive Assignment of Linear Secondary Structure Elements) **that delineates secondary structure elements (SSEs) from protein C_α _coordinates and specifically addresses the requirements of vector-based protein similarity searches. Our program identifies two types of secondary structures: helix and β-strand, typically those that can be well approximated by vectors. In contrast to traditional secondary structure algorithms, which identify a secondary structure state for every residue in a protein chain, our program attributes residues to linear SSEs. Consecutive elements may overlap, thus allowing residues located at the overlapping region to have more than one secondary structure type.

**Conclusion:**

PALSSE is predictive in nature and can assign about 80% of the protein chain to SSEs as compared to 53% by DSSP and 57% by P-SEA. Such a generous assignment ensures almost every residue is part of an element and is used in structural comparisons. Our results are in agreement with human judgment and DSSP. The method is robust to coordinate errors and can be used to define SSEs even in poorly refined and low-resolution structures. The program and results are available at .

## Background

Protein secondary structure was first predicted on the basis of stereo-chemical principles to adopt α-helical and β-strand conformations due to the periodicity of their inter-backbone hydrogen bonds [1]. Subsequent experimental determination of protein structures confirmed these predictions revealing the presence of α-helices and β-strands as the predominant secondary structure elements (SSEs) in proteins. Other minor SSEs such as 3_10_-helix [2], π-helix [3], β-turns (type I-IV) [4], γ-turns [5], and β-bulges [6] have been defined based on the stereochemistry of the polypeptide chain. However, deviations from ideal geometry in experimentally determined structures due to interactions between the elements and possible errors in coordinates can make an algorithmic definition that is consistent with manual assignment challenging.

The first algorithm for the automatic delineation of secondary structure was proposed by Levitt and Greer [7]. They defined secondary structures based on peptide hydrogen bonds (using i, i+3 C_α _distances and i, i+1, i+2, i+3 C_α _torsion angles). A more comprehensive algorithm, DSSP, was subsequently developed and is based on a careful analysis of backbone-backbone hydrogen bond energies and geometrical features of the polypeptide chain [8]. It is very accurate in its residue-based definition of the available coordinates and does not attempt to interpret the data in any predictive way. Another well-known secondary structure definition program is STRIDE [9]. Like DSSP, this program also uses hydrogen bond energy as well as main chain dihedral angles φ and ψ. In addition, it relies on a database of derived recognition parameters that uses the crystallographer's definition of secondary structures as the standard. Other programs to assign the secondary structure states of proteins exist. Xtlsstr defines secondary structure in the same way a person assigns secondary structure visually [10]. It uses two angles and three distances computed from the protein backbone atoms. Sstruc is a reimplementation of the classic DSSP method (Smith DK, Thornton J; unpublished). The P-Curve algorithm allows the helicoidal structure of a protein to be calculated starting from the atomic coordinates of its peptide backbone [11].

Secondary structures in experimentally determined protein coordinate data often deviate from the ideal geometry and thus methods of secondary structure assignment that use different logic and cutoffs can vary significantly in their assignments. Maximum variation is seen near the edges of SSEs and consensus secondary structures defined by different algorithms have been proposed in order to define SSEs accurately [12]. Attempts have been made to define secondary structures consistently and in agreement with visual inspection by recognizing errors in protein coordinates. In the Stick algorithm, line segments become the primary data elements and can then be used to define secondary structure. By contrast, previous approaches have used secondary structure definitions to specify line segments [13]. Our algorithm retains the former approach, as C_α _geometry allows locating breakpoints in both α-helices and β-strands and allows generation of residue based pairing information helpful in determining edges of β-sheets.

A strong correlation exists between hydrogen bonding patterns and C_α _distances and torsion angles [14]. Algorithms such as those described by Levitt [7], DEFINE_S [15], VOTAP [16] and P-SEA [17] assign secondary structure not on the basis of actual hydrogen bonding patterns, but using an interpreted residue pairing based on the C_α _coordinates of the protein structure. However, most of these programs assign secondary structure properties to individual residues of a protein chain. For the purpose of vector-based structural similarity searches, a secondary structure definition of the linear segments (elements) that can be used to approximate the protein structure in a simplified form as a set of interacting SSEs is required. Using DSSP [8] assignments to define SSEs for protein structure comparisons may lead to problems in identifying linear elements and element edges [18]. Our analysis of DSSP assignments reveals that they are not well adapted to the definition of SSEs for several reasons. DSSP finds a secondary structure state for every individual residue in a protein chain. When these states are found, the consecutive residues that belong to one state can be unified to form a SSE. Such a strategy of element definition has several disadvantages. First, one might wrongfully unify two consecutive elements into one. Alternatively, if there is a gap in the secondary structural state of a residue inside an element, due to disorder, refinement error or some other irregularity, this element will be unjustifiably split into two. Residue coverage for DSSP assignments is poor when the structure is not well refined or not well ordered and the stringent criteria set for hydrogen bonds are not met. DSSP misses some short helices and β-strands in which hydrogen bonding criteria are violated. Moreover, the edges of elements are not always defined accurately. Thus, although DSSP can identify a secondary structure state as a property of each residue, it not well suited to outline SSEs.

Here we describe a method "Predictive Assignment of Linear Secondary Structure Elements (**PALSSE**)" to identify SSEs from the three-dimensional protein coordinates. The method is intended as a reliable predictive linear secondary structure definition algorithm that could provide an element-based representation of a protein molecule. Our algorithm is predictive in that it attempts to overlook isolated errors in residue coordinates and is geared towards defining SSEs of proteins relevant to vector-based protein structure comparison. For the purpose of similarity searches, use of just the major SSEs, namely the α-helix and the β-strand that can be approximated by vectors will suffice, as they typically incorporate the majority of the residues in a protein.

Our program delineates α-helices, and β-strands participating in β-sheets. Helices are broadly defined to include right-handed α-helices, 3_10 _helices, π-helices and turns that show a helical propensity. We do not distinguish between these helices since many linear helical elements in proteins combine 2 or more helical types. For example, the first turn of a α-helix might be a 3_10 _helix and the last turn could be a π-helix. If one would like to differentiate between the three types of helices, DSSP [8] or STRIDE [9] should be used. Similarly, the β-strand elements are broadly defined by our program to include β-strands, β-bridges, β-bends and the residues of β-hairpins.

## Results and discussion

### Brief overview of the algorithm

α-helices and β-strands are the predominant and most distinct types of secondary structures observed in proteins [8,9,19]. Using only C_α _coordinates, our algorithm delineates two types of secondary structural elements, namely helices (includes α, 3_10 _and π-helices) and β-strands (includes β-strands, β-bridges, β-bends and the residues of β-hairpins).

Our method was developed for predictive assignment of linear SSEs. Preliminary helix and β-strand categories are assigned to residues, based on i, i+3 C_α _distance and i, i+1, i+2, i+3 C_α _torsion angle (step 1 in methods). Next, probable helix and β-strand elements are generated by selecting consecutive residues that belong to the same category (helix or strand, step 2). Quadruplets of residues, formed by two pairs of hydrogen-bonded consecutive residues that satisfy criteria of distances and angles (steps 3 and 4), are constructed from residues that do not meet the strict criteria for helix definition in step 1. A quadruplet is the smallest unit for defining potential β-sheets and is formed from a set of four C_α _atoms that are linked with two covalent bonds and two pseudo-hydrogen bonds (see step 3 in methods). The quadruplets are joined together, end-to-end in the direction of covalent bonds, to form ladders of consecutive pairs of residues (steps 5, 6, 7). The ladders of paired residues are joined to form paired β-strands. Helices defined previously are split, using root mean square deviation (RMSD) of constituent residues about the helix axis, so that they can be represented as linear elements (step 8). β-strands are split using various geometrical criteria and pairing of neighboring residues (step 9).

The program's main output is in the PDB [20] file format. HELIX and STRAND records are added or substituted by our definition.

### Defining and extending core α-helices and β-strands

**Figure 1:**
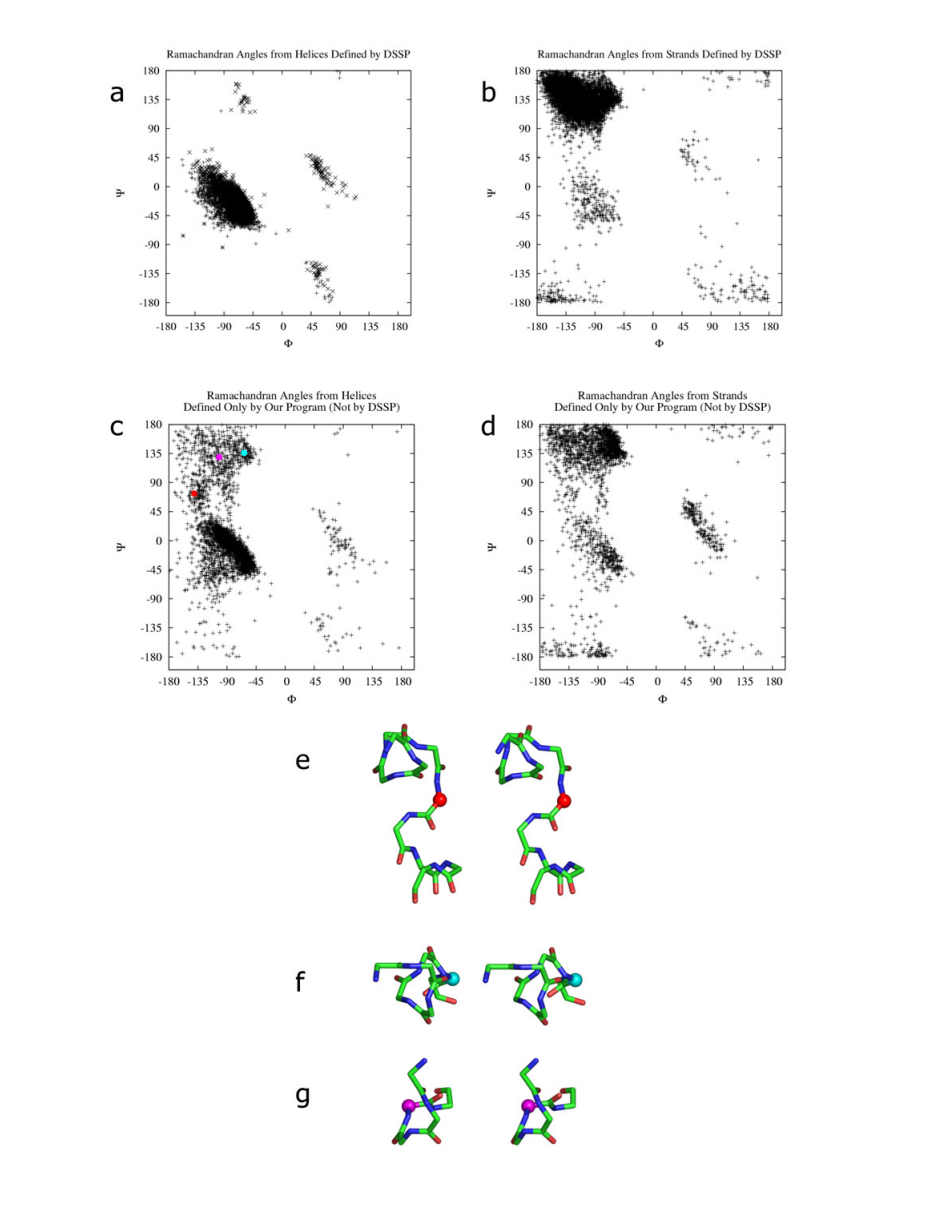
Ramachandran angles from helices and β-strands defined by DSSP and our program. The culled PDB set (described in methods) was used for this calculation. Figs. a and b show the Ramachandran angles obtained respectively from helices and β-strands defined by DSSP. Figs. c and d show the Ramachandran angles obtained respectively from helices and β-strands not defined by DSSP but defined by our program. φ and ψ angles for figs. a and b were obtained from DSSP output. φ and ψ angles for figs. c and d were calculated from output of our algorithm such that φ is torsion angle between residues i-1 and i, and ψ is torsion angle between residues i and i+1 where residues at positions i-1, i and i+1 are part of the same SSE. α, π and 3_10 _helices were used for obtaining data shown in fig. a (DSSP definition 'H', 'I', 'G' respectively). β-Strands were used for obtaining data shown in fig. b (DSSP definition 'E'). Three regions of over-predicted points by our method are shown with an example from each region. Figs. e, f and g show stereo diagrams of parts of three helices respectively from "1b × 4" (chain A, residue 175 in red), "1iom" (chain A, residue 73 in cyan) and "1 × 7d" (chain A, residue 180 in magenta). φ and ψ angles from the residues under study are marked red, cyan and magenta in fig. c. The residues with φ and ψ points highlighted in fig. c are shown as spheres in the same colors in figs. e, f and g.

C_α_-C_α _distance (i, i+3) and torsion angle (i, i+1, i+2, i+3) are used to select core regions of secondary structures. The parameters are then made less restrictive to identify and assign residues that do not follow the idealized pattern of α-helices and β-strands. Residues that individually might fail the test for a secondary structure state, due to either hydrogen-bonding criteria or φ and ψ angles, or both, may be placed in an element if the geometric parameters and pairing conditions of the neighboring residues support the inclusion. This makes the algorithm predictive in nature; therefore helices and β-strands might be defined in regions that show a helical tendency or have neighboring β-strands respectively, even if the polypeptide model at that region is erroneous. We have found the criteria of a minimum of 3 residues with at least 2 residues pairing with a neighboring β-strand [7] to perform well in identifying β-sheets that match human judgment. Thus, anti-parallel pairs of β-strands with two residues each never arise from loop regions. A method based on quadruplets of residues linked with two covalent bonds and two hydrogen bonds, approximated using a set of three parameters based on distances and angles between the residues, have been used as the seed unit for β-sheets. A quadruplet-based approach has also been used by Levitt [7]. β-strands are defined only when one or more neighboring paired β-strands are available, thus reducing the chance for errors in our predictive definition of β-strands. The smallest helix by our definition has a length of 5 residues, which is a single turn of a α-helix. Our algorithm assigns small 5-residue turns showing helical propensity as helices. Other programs that are C_α_-based also fail to distinguish between such turns and helices [21]. As shown in fig. 1a, only a few of these assignments are actually incorrect.

### Definition of linear elements

Our method attempts to provide a definition of SSEs that can be approximated using vectors. It is possible for edges of elements to overlap, leading to more than one secondary structure state for a particular residue. If required, these elements are broken using geometric criteria and directionality changes over a stretch of residues, including pairing for β-strands, to obtain linear elements. Specialized algorithms to detect curved, kinked and linear helices based on local helical twist, rise and virtual torsion angle are known [22]. We have used simpler rule-based methods to improve computational speed. Single residues existing between two bent helices fail cutoffs for C_α _distance and torsion angles. Bent helices thus remain separate even though their edges might overlap. Gently curved helices are not broken unless the angle between vectors representing the broken sections is greater than 20°. The broken sections are chosen using an approach (described in methods) to minimize the number of acceptably linear elements, while retaining the maximum number of residues from the original helix. A similar breaking angle of 25° has been observed by Richards and Kundrot [15]. This angle and RMSD are used to break kinked helices correctly. Kinked helices, however, are retained if the angle between their representative vectors is less than 20°.

### Reliability of secondary structure definitions

The reliability of secondary structure definition by our algorithm was checked by plotting the main chain torsion angles φ and ψ [23], individually for all helix and β-strand regions defined by DSSP [8] (figs. 1a, 1b) and defined only by our program when compared with DSSP (figs. 1c, 1d). Examples of helices (fig. 1c) and β-strands were inspected manually.

Most of the φ and ψ angles (fig. 1c) are found to be in the regions of the Ramachandran plot commonly accepted to be representative of α-helices [23,24]. An example from the region bounded by -150°<φ-125° and 50<°ψ<100° is shown as a red dot in figs. 1c and 1e. Residues with φ and ψ angles in this region occur mostly at overlaps between two helix elements. Residues with φ and ψ angles in the region -50°<φ<-75° and 100°<ψ<150° (shown as a cyan dot in figs. 1c and 1f) are mostly edge residues of helices. Secondary structure for residues with φ and ψ angles in the -100°<φ<-125° and 125°<ψ<150° region (shown as a magenta dot in figs. 1c and 1g) are not immediately obvious. These are mostly at the edge of helices and could potentially be part of a β-strand or extended region. However, for the examples that we studied, it would not be wrong to define these residues as belonging to part of a helix as no consecutive β-strands were present and the edge residues showed helical tendency. The region of the plot at 50°<φ<100° and -50°<ψ<50° consists of residues taking part in 3_10_-helices.

Nearly all φ and ψ angles for β-strand residues over-predicted by our method (fig. 1d) fall in the same regions as that of DSSP [8] defined β-strands, and most were found to fall in the commonly accepted region of the Ramachandran plot for β-strands [23,24]. Examples of β-strand residues with φ and ψ angles outside this region were inspected manually. A large number of residues with angles in the region -100°<φ<-50° and -50°<ψ<0° were found to be at overlaps between helices and β-strands. Residues with angles in the region 50°<φ<100° and -50°<ψ<50° are mostly bulges and regions of overlap between two β-strand elements. Some residues were found to be included in our element definition but would not be assigned a secondary structure by a residue-based method. These had angles in the region 50°<φ<150° and -180°<ψ<-125° and also 50°<φ<150° and 150°<ψ<180°. These residues are rare in β-strands and were not found to occur successively. They sometimes form part of bulges or element edges.

Residues that fail strict secondary structure assignment when explicit hydrogen-bonding criteria are considered, cannot be used to properly form SSEs. Therefore, predictive assignment with our algorithm is preferred. The following reasons may account for the necessity of predictive assignments. Protein structures are intrinsically flexible. Hydrogen bonds that are present in some family members might be absent in other homologues. Domain interactions, loops, insertions and deletions can all influence the secondary structure around them. Crystal packing and solvent interaction can also account for changes observed in residue coordinates. Further, models based on X-ray data are not always as accurate as they are believed to be [25]. Therefore, the absence of a strictly defined hydrogen bond does not mean that the hydrogen bond is actually absent in the molecule in all its accessible conformations.

### Robustness towards coordinate errors

**Figure 2:**
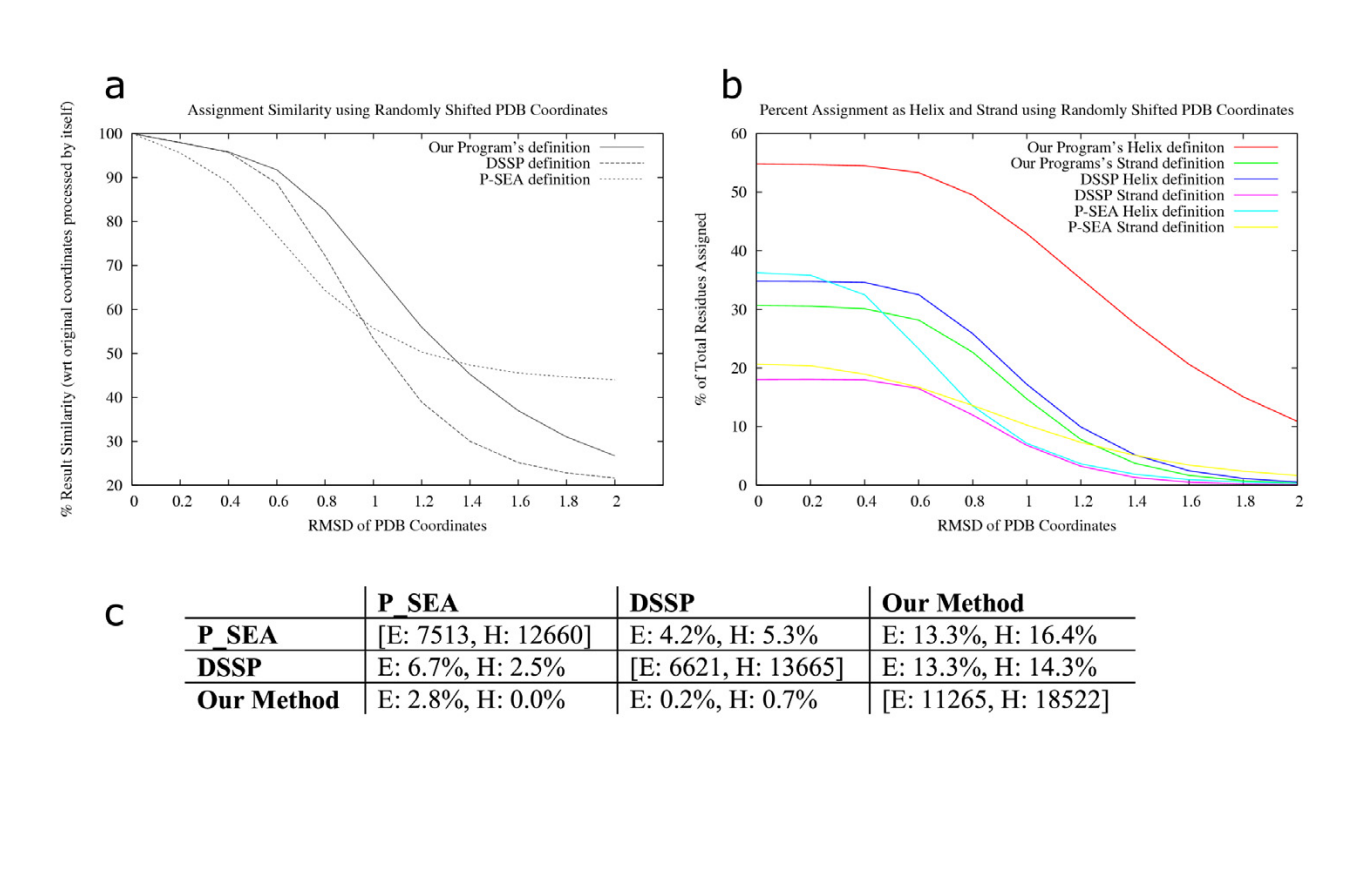
Secondary structure assignment reliability for DSSP, P-SEA and our program using randomly shifted PDB coordinates. The culled PDB set (described in methods) was used for this calculation. Gaussian random numbers were used to randomly shift coordinates of residues from 0.2Å to 2Å in steps of 0.2Å, in the PDB files. 100 files were generated for every file for every data-point leading to a total of 1,00,000 randomly shifted coordinate files. 2a: Mean and standard error of assignment consistency compared with assignment by the same program on the original coordinates. A percentage match was calculated by comparing definitions for the coordinate shifted file with the program output from actual file on a per residue basis. Means for the percentage match are shown. Standard errors were about 1% in each case (not shown). 2b: Average secondary structure content defined by each program for PDB files at different levels of perturbations are shown. The files used are the same as for fig. 8a. The number of residues assigned as helices or β-strands are shown as a percentage of total residues. Spaces and coils in the program output are counted for calculating percentages. 2c: Percentage of residues over-predicted by each program (DSSP, P-SEA, our method) with respect to the other two is shown. 100 files from the culled PDB set were used for these calculations. 35,670 residues were considered. Results shown are for over-predictions by program names in the column heads when compared with program names in the row heads. Actual number of helices and β-strands assigned by the program are shown on the diagonal (bracketed values).

Our algorithm shows a higher degree of robustness than either DSSP [8] or P-SEA [17] when input data contains errors of up to 1.5Å deviation for individual residues (fig. 2a). Secondary structure definition, by DSSP, using main-chain atoms deteriorate sharply when coordinates are non ideal for α-helices or β-strands. Other methods based on recurrence quantifications have been proposed [26] that aim to replicate DSSP definitions while being more robust to coordinate errors. These results are meaningful only at high residue coverage. Our algorithm is able to delineate more helical regions, than DSSP or P-SEA, even when the coordinates are randomly shifted by as much as 2Å (fig. 2b). Close to 80% of all residues are assigned to SSEs by our method. Defining a core of strict α-helix and β-strand forming regions and extending these with neighboring residues to obtain elements ensures that residues are not lost from SSEs when they fail strict definitions, like in low-resolution X-ray and NMR structures.

Keeping the above results in perspective, the amount of secondary structure missed by the two other programs with respect to each of DSSP, P-SEA and our method was studied (fig. 2c). Our program is able to assign about 14% more residues as helices and 13% more residues as β-strands when compared with either DSSP or P-SEA. DSSP assigns less than 1% residues that differ from our definition. P-SEA assigns less than 3% residues that differ from our definition. Most residues defined by DSSP but not assigned by our algorithm did not contribute to long SSEs.

### Comparison with other programs

Definitions from our program were compared with assignments by DSSP [8], P-SEA [17], DSSPCont [27], SSTRUC (Smith DK, Thornton J; unpublished), STRIDE [9], DEFINE_S [15], STICK [13] and PROSS [28]. A numerical comparison of residue assignment between secondary structure delineation methods, irrespective of whether they define elements or are residue-based, is not very useful. Element lengths may not be optimum or elements may be too curved for representation by vectors. Residues assigned to α-helix or β-strand regions may not overlap between different programs, thus leading to a wrong comparison if only numbers are used.

**Figure 3:**
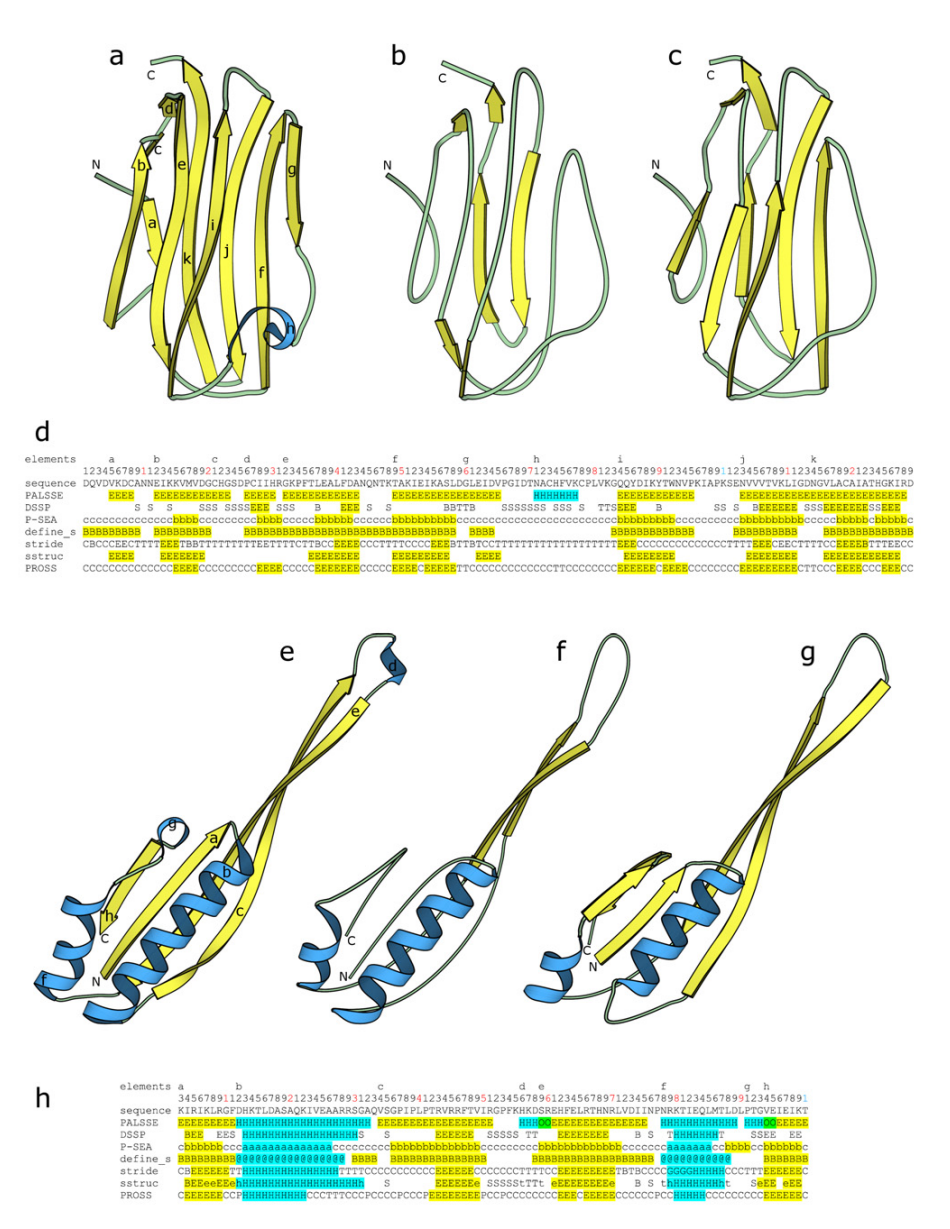
Two examples of secondary structure assignment by different programs. We chose an averaged NMR structure "1ahk" and a low-resolution X-ray structure (3.0Å) "1fjg" to show as examples since over-prediction by our method is maximum for such structures. Only chain "J" of "1fjg" is shown. Figs. a, b, c show cartoon diagrams of "1ahk", prepared using MOLSCRIPT [46], for β-strand and α-helix definitions by our program, DSSP [8] and P-SEA [17] respectively. β-Strands are shown in yellow and helices in cyan. The N- and C- termini are marked for each structural diagram. The elements produced by our program are labeled. Fig. d shows the secondary structure assignment for "1ahk" by PALSSE (our program), DSSP, P-SEA, DEFINE_S, STRIDE, SSTRUC and PROSS. Our interpretation of β-strands and helices as defined by the different programs are colored in yellow and cyan respectively. The starting positions of each element labeled in fig. a are shown on the first line. The sequence is numbered on the second line with black letters denoting units, red denoting tenths and blue denoting the hundredths places. The protein sequence is shown in the third line. Figs. e, f, g shows cartoon diagrams of chain "J" of "1fjg", prepared using MOLSCRIPT [46], highlighting β-strand and helix definitions by our program, DSSP, and P-SEA respectively. β-Strands are in yellow and helices are shown in cyan. Elements are labeled in fig. e. Fig. h shows the secondary structure alignment for "J" chain of "1fjg". Definitions produced by the same programs as that used for fig. d are shown. Yellow color is used for our interpretation of β-strands and cyan denotes our interpretation of helices. Green has been used to denote overlaps between helix and β-strand elements defined by our program. The first, second, and third lines show start of each element in fig. e, residue number and sequence respectively, similar to fig d. DSSP, P-SEA, DEFINE_S, SSTRUC, STRIDE and PROSS assignments were generated by obtaining the programs, and then compiling and running them with default parameters on the example PDB files.

In this article, we show two examples of our study (fig. 3), with the secondary structure definitions colored cyan or yellow respectively for helices and β-strands. Two-residue β-strand definitions have not been considered as β-strands for this comparison. Figs. 3a–d are obtained by processing the coordinates of an averaged NMR structure (PDB ID: 1ahk [29]) containing an immunoglobulin sandwich made up of 7 β-strands in two β-sheets. Figs. 3e–h are from a low-resolution (3.0Å) X-ray structure (PDB ID: 1fjg, chain "J" [30]) which has a ferredoxin-like fold made up of two β-α-β units.

Our algorithm shows a marked difference as compared to other programs when low-resolution and NMR structure coordinates are processed. Residue coverage is greater for our definition when compared with DSSP [8] and P-SEA [17], and is also correct with respect to identified elements when used for a similarity search. Fig. 3a shows that our method has been able to correctly identify all 7 β-strands of the immunoglobulin fold, compared to only 2 by DSSP (fig. 3b) and 6 by P-SEA (fig. 3c). Our method has also been able to correctly identify all 4 β-strands and 2 helices in the ferredoxin like fold (fig. 3e), compared to only 2 α-helices and parts of 2 β-strands by DSSP. A vector-based similarity search system will produce incorrect results with the DSSP assignments shown, as the β-strands will be found to be located too far away. Assignment by P-SEA shortens the helix and incorrectly adds a β-strand to the definition. Our interpretation of helix and β-strands from PDB files 1ahk and 1fjg (chain "J") by various programs are respectively colored in figs. 3d and 3h. It is interesting to note the wide variation in assignments of secondary structures by different programs. The only other program that compares favorably in residue coverage to P-SEA and our program is DEFINE_S. However, DEFINE_S sometimes misses helices and wrongly defines parts of helices as β-strands. We have not found the DSSPCont [27] output to be any different from that of DSSP, unless the results are interpreted using probability scores in the output file. STRIDE [9] results are not significantly different from that of DSSP for the examples shown, even though the STRIDE algorithm attempts to improve upon DSSP definitions using a common criterion of hydrogen bonding energy as well as C_α _distances. Results from SSTRUC (Smith DK, Thornton J; unpublished) are clearly different and residue coverage is poor when compared with other programs for the average NMR structure "1ahk". Residue coverage and element identification for SSTRUC is similar to that by DSSP and STRIDE for "1fjg". Residue coverage for helix and β-strand definition by PROSS [28] is low and it fails to identify all elements.

## Conclusion

The algorithm developed by us can assign linear helix and β-strand SSEs, from only C_α _atoms. Our method is predictive in nature and SSEs defined by us can include residues that do not form ideal α-helices and β-strands. Assignments are similar to helix and β-strands defined by a residue-based approach, like DSSP [8], for high-resolution X-ray structures, although our elements include more edge residues. For NMR and low-resolution X-ray structures, our predictive algorithm delineates more SSEs than is possible with a residue-based approach. Elements defined by our method can be approximated using vectors, and we have retained longer elements wherever possible.

This method has been developed for simplified representation of protein structures for similarity searches with other proteins. It should not be used if an accurate residue level definition is necessary. Compared to other programs, we have found our algorithm to perform well in terms of defining linear elements reliably for both helices and β-strands and yet yield a high residue coverage. Visual judgment of results supports our definitions.

The algorithm has been implemented as a computer program "PALSSE" (Predictive Assigner of Linear Secondary Structure Elements). It is written in Python and C and has been tested on the GNU/Linux platform on the i386 processor architecture. The software is available online [31].

## Methods

### Datasets used for generation of statistics and for expert judgment

The sequences from the SEQRES records of PDB [20] files, current until March 2002, and solved using X-ray diffraction (resolution better than 4.0 Å), and NMR techniques were clustered using BLASTCLUST [32] to obtain clusters with sequence similarity less than 25% and overlap greater than 90%. After screening the resulting chains for errors, format violations, and cases that would prevent either DSSP [8] or our program from processing the file, we obtained a dataset of 2787 polypeptide chains (Statset). This set was used to determine appropriate cutoff values for various parameters in our program.

Algorithm development was monitored by manual inspection of the results produced by the implemented code. For this, a dataset of 295 domains (checkset) consisting of randomly chosen representative structures for every fold in the SCOP database (version 1.63) [33,34] belonging to the 'all α proteins' and 'α and β proteins' classes were chosen.

A set of high-resolution structures (culled PDB set) was used for comparing the final program output with that from other programs, with respect to reliability and robustness towards coordinate errors. For this, a list of the 100 longest non fragmented PDB chains having resolution better than 1.6Å and sequence similarity less than 20% were obtained from the culled PDB database [35].

### Programming platform

We used the Python programming language [36] (v2.3) to implement our algorithm. We also used the Biopython [37] Bio. PDB [38] framework to parse the PDB [20] files and store data in its internal data structures. This is a robust method of parsing PDB files and can satisfactorily handle many common problems with parsing NMR models, multiple chains, alternate locations and insertion codes. The "Polypeptide" module enabled handling of chain segments. The included functions were used to store and retrieve data from the constituent objects. Our project extends the Biopython framework to define secondary structures. The implemented code was tested on GNU/Linux [39] systems on the i386 and opteron hardware running Linux i386 kernel. All data plots shown in this article have been prepared using "gnuplot" [40]. All protein structure images were prepared using PyMOL [41] and POV-Ray [42] unless specified otherwise.

### Brief overview of methodology

Our method has five major steps that are used to sequentially process the PDB [43] coordinates and assign secondary structure. These are: 1: Assignment of helix and β-strand propensities to individual residues based on i, i+3 C_α _distance and i, i+1, i+2, i+3 C_α _torsion angle. 2: Delineation of probable core regions of helix and β-strand elements from residues that pass strict criteria. 3: Formation of quadruplets of residues connected by two covalent and two hydrogen bonds as seeding units for β-sheets. 4: Initiation and extension of paired β-strands using quadruplets of paired residues. 5: Breaking consecutive non-single helices and β-strands taking into consideration residue pairing and neighboring elements. Steps of our algorithm need to be run sequentially for the results to be meaningful.

### Step 1: Assignment of helix and β-strand propensities to individual residues

**Figure 4:**
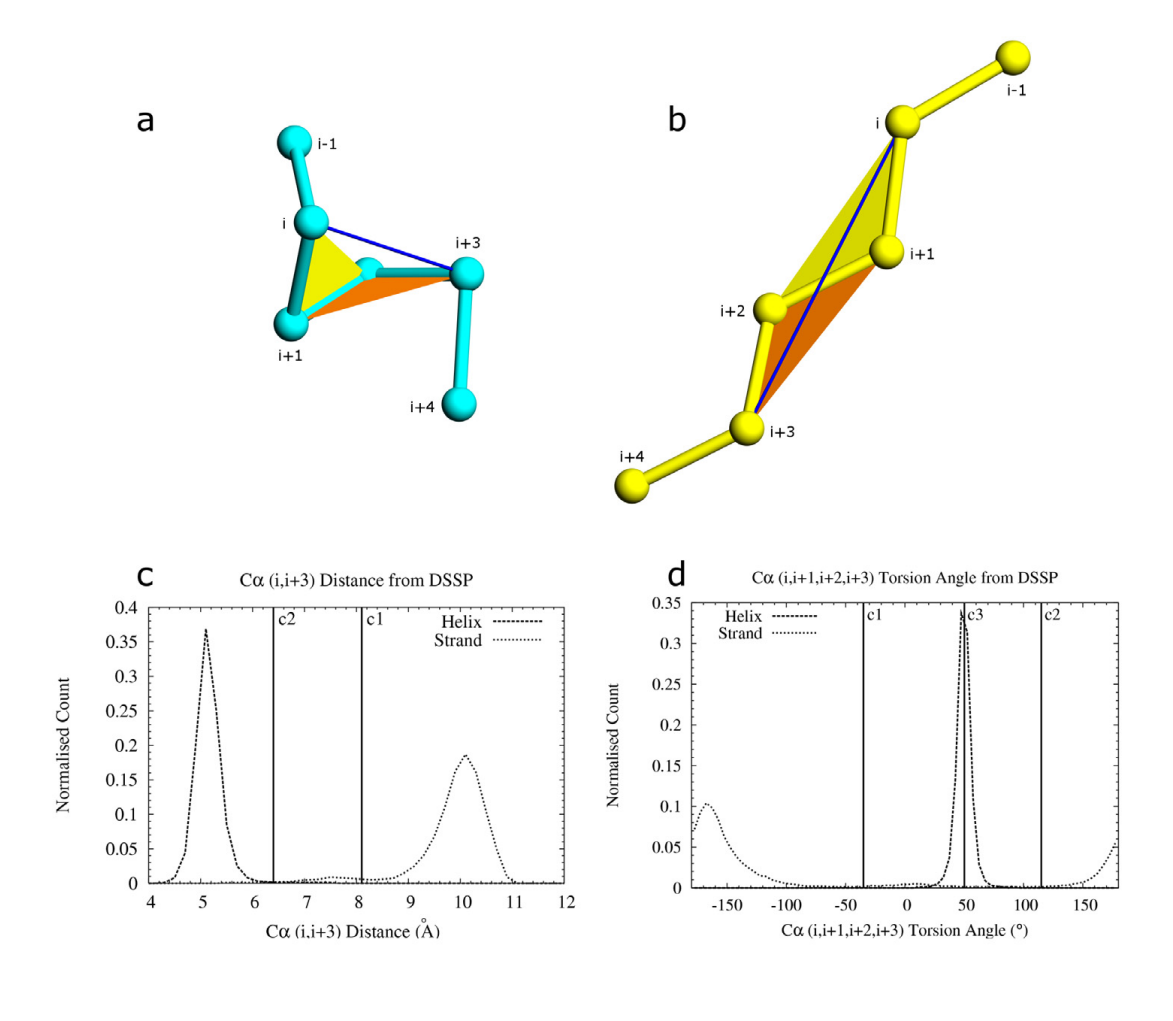
Parameters for assignment of helix and β-strand property to individual residues. C_α _distance and C_α _torsion angle were calculated for defining helices (fig. a) and β-strands (fig. b). The distance between residues i, i+3 (shown joined by a blue line) and torsion angle between residues i, i+1, i+2, i+3 (shown as angle between two colored planes; yellow plane between residues i, i+1, i+2 and orange plane between residues i+1, i+2, i+3) are used to assign loose-helix, strict-helix and loose-strand secondary structure property to individual residues. 4c: Distance between i, i+3 C_α _residues from helix and β-strand definitions obtained from DSSP [8] output. Distances were binned in 0.2 Å intervals. Cutoff distance c1 (8.1 Å) is the maximum distance allowed for assigning loose-helix property to a residue. Cutoff c1 is also the minimum distance allowed for assigning loose-strand property to a residue. Residues at i and i+3 positions are allowed in the same SSE template (SSET) only if the cutoff distance c1 passes. Cutoff c2 (6.4 Å) is the maximum i, i+3 C_α _distance for strict helix definition. 4d: Torsion angle between i, i+1, i+2, i+3 C_α _atoms for helix and β-strand definitions obtained from DSSP output. Angles are binned in 5° intervals. A loose-helix definition is assigned to a residue only if the torsion angle for the residue falls between c1 (-35°) and c2 (115°). A loose-strand is assigned only if the torsion angle is -180° to c1 or c2 to 180°. c3 is the optimal torsion angle for helices and is used to define strict-helix residues if the torsion angle is within a 2 sigma deviation from c3.

Our algorithm first conservatively estimates the propensity of each residue to be part of a secondary structural state (helix, β-strand, both, or none). This is the only step in which our algorithm deals with secondary structure as a property of the individual residue and not as that of an element. C_α _coordinates of every residue of the molecule are processed from the N- to C- terminal end and a simple C_α_-C_α _(i, i+3) distance (fig. 4a) and C_α _torsion angle (i, i+1, i+2, i+3) (fig. 4b) are used to mark residues based on whether they have a propensity to assume helical or β-strand conformation. It is possible for a residue to be part of both a helix and a β-strand, or none of them (coil). We decided to use i, i+3 C_α_-C_α _distances and i, i+1, i+2, i+3 C_α _torsion angles as these parameters have been studied extensively [7,8,15,21] with regards to their applicability to secondary structure definition.

The output of DSSP [8] was used to generate cutoff statistics for initial residue-based definition, since it is a conservative program that delineates secondary structures based on hydrogen bonding energy. i, i+3 C_α_-C_α _distances (fig. 4c) and 4i, i+1, i+2, i+3 C_α _torsion angles (fig. 4d) were calculated separately for α-helices and β-strands from DSSP output generated from the "Statset". In this calculation, only residues corresponding to the letters 'H' and 'E' in the 'structure' column of DSSP output were considered as helices and β-strands respectively. π, 3_10_-helices, and β-turns were not used as we decided to initiate secondary structural elements conservatively, using these cutoffs, and then extend them rather than to start from spurious elements for later removal.

DSSP [8] provides assignment for individual residues and does not assemble them into SSEs. We used DSSP data to generate elements, and slightly different approaches were taken for α-helices and β-strands. All consecutive residues belonging to the helical state were considered as part of the same α-helix. For β-strands, DSSP provides bonded pair information (column 'BP1' and 'BP2' in DSSP output) and this was used to locate continuous stretches of paired residues. Each set of these continuous residues was considered as an element. Distance and torsion angle data were calculated from the 'N-' to 'C-' terminal end of the molecule. The data was used to determine cutoff conditions for identifying potential helix and β-strand-rich regions in this step of our algorithm. The following functions were used to decide a particular residue's propensity to form part of helix or β-strand.

δ<8.1 Å, -35°≤τ≤115° ⇒ ρ=*loose-helix *    (1)

δ>8.1 Å, -180°≤τ<-35° | 115°<τ≤180° ⇒ ρ=*loose-strand *   (2)

δ≤6.4 Å, τ *within *(50.1° ± 2σ) ⇒ ρ=*strict-helix *   (3)

where δ is C_α_-C_α _distance, τ is C_α _torsion angle (i, i+1, i+2, i+3), σ is standard deviation of torsion angle (= 8.6°), ρ is propensity (cutoff values from figs. 4c, d).

Strict-helix residues thus form a subset of the loose-helix residues. This eliminates β-bends from interfering with helix definition even though it also removes π-helices, which have a greater i, i+3 C_α _distance than α-helices. Left-handed helices are also removed as they have a lower i, i+1, i+2, i+3 C_α _torsion angle than α-helices.

The secondary structure definitions at the end of this step are in agreement with the findings of several other groups who have interpreted secondary structures from just the C_α _coordinates [7,15,17,21]. In our program, we use this step as an initial criterion for defining secondary structure states of individual residues. This definition is further refined and extended in subsequent steps of our program to define SSEs. Estimation of secondary structure of individual residues is an important step of the algorithm, however the rest of the algorithm is also designed to correct for errors that may have been introduced at this step.

### Step 2: Delineation of probable helix and β-strand elements

Consecutive residues with the same secondary structural propensity are joined to initiate seed-SSEs. Overlapping elements are considered in this step. The overlap can be between elements of the same (H-H, E-E) or different type (H-E, E-H). Since we always process PDB [20] files from the N- to the C- terminal end of the polypeptide for generating cutoff data and also generating C_α _distance and torsion angles for this algorithm, extension of seed-SSEs using the distance and torsion angle data was done from the C-terminal end of the seed-SSE towards the C- terminal end of the polypeptide.

We use a group of at least two consecutive loose-helix residues to generate a loose-helix seed-SSE. It is possible to get a single loose-helix residue in β-hairpins whereas a set of consecutive loose-helix residues is more likely to be a part of a helix as it signifies that four (i, i+1, i+3, i+4) residues out of a five residue group (i – i+4) have passed cutoffs for i, i+3 C_α _distance and i, i+1, i+2, i+3 C_α _torsion angle. Loose-helix SSE templates (SSE template henceforth referred to as SSET) consisting of at least five residues are formed from every set of consecutive loose-helix residues and the three residues immediately succeeding them. This process ensures that a helical element is generated from only a single continuous region of loose-helix residues. At this stage, it is possible that the third residue of a five residue loose-helix SSET has not passed the cutoffs for distance and torsion angle with any other residue. Strict-helix seed-SSE is defined by a group of three consecutive strict-helix residues. Strict-helix SSETs are formed from a strict-helix seed-SSE and the three residues immediately after it. This implies that every residue in a strict-helix SSET passes the strict cutoffs of distance and torsion angle with at least one other residue making the minimum length of a strict-helix SSET six residues. All loose-helix SSETs that do not contain at least one strict-helix residue, other than in the last three residues, are discarded. The remaining loose-helix SSETs denote possible helix templates.

A loose-strand-forming seed-SSE is defined by a group of loose-strand residues, with at least one residue in the group. Extending every loose-strand seed-SSE to the i+3 position at the C- terminal end gives rise to loose-strand SSETs. Overlaps between elements are formed during extension of the C- terminal end of the seed-SSE to the i+3 residue leading to a maximum overlap of two residues between any two SSETs.

### Step 3: Quadruplets as seeding units of paired β-strands

**Figure 5:**
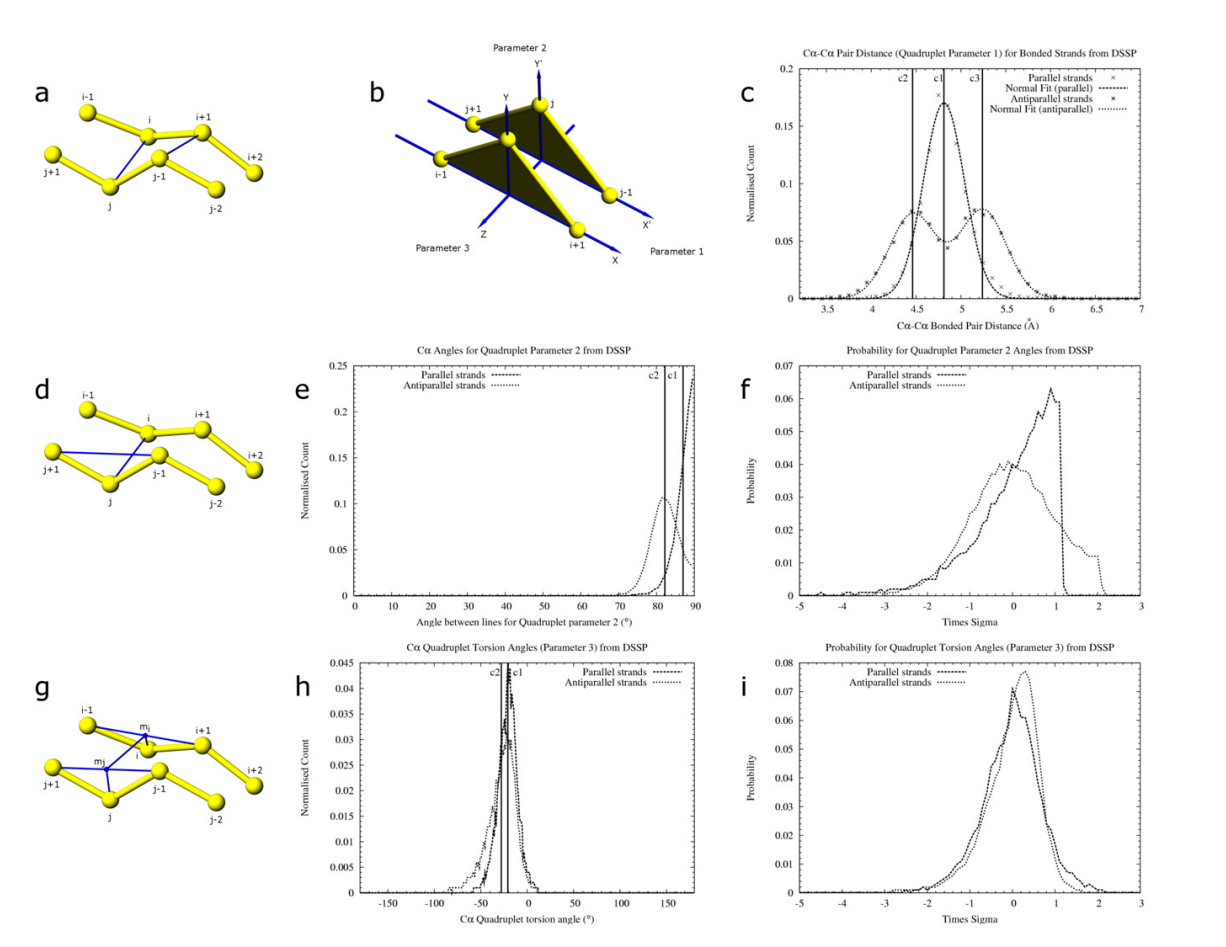
The three parameters on which quadruplets scoring is based. In figs. a, d, g, a quadruplet is formed from residues i, i+1, j-1 and j. The score is used to select the best quadruplets to join and form β-sheets. Each scoring parameter has been chosen such that they least influence each other. Residues i-1, i+2, j+1 and j-2 are required to calculate angles for quadruplet scoring. The first parameter is C_α_-C_α _distance between paired residues (fig. a). Blue lines joining i, j and i+1, j-1 show the distance being scored. This parameter approximates the deviation of the triangle apex i with reference to the triangle apex j in fig. b due to rotation of the plane i-1, i, i+1 on the X axis. Fig. c shows the C_α_-C_α _distances for parallel and antiparallel β-strands obtained from DSSP [8] output. Data is binned at 0.1 Å intervals and fit to a normal distribution using "gnuplot" [40]. Distribution for parallel β-strands has a mean at c1 (4.81 Å) with a sigma of 0.22. Distance for antiparallel β-strands follows a bi-modal distribution with means (μ) at c2 (4.46 Å) and c3(5.24 Å) and a standard deviation (σ) of 0.26. These μ and σ values were used to calculate the probability of occurrence of C_α_-C_α _pairing distances while scoring quadruplets by our algorithm. A C_α_-C_α _maximum distance of 7.5 Å (not shown) was used to limit pairing between residues. The second parameter is angle between lines (shown in blue) joining the vertices i, j and the base j-1, j+1 of the imaginary triangles j-1, j, j+1 and i+1, i, i-1 (fig. d). Only one of the four cases is shown. The other angles are between lines j, i and i+1, i-1; j-1, i+1 and i, i+2; i+1, j-1 and j, j-2. Deviation of this angle approximates the deviation of the triangle apex i-1 with reference to the triangle apex j+1 in fig. b due to rotation of the plane i-1, i, i+1 on the Y axis. Fig. e shows the distribution of angles, binned at 5° intervals, obtained from parallel and antiparallel β-strands defined by DSSP where c1 (87°) and c2 (82.2°) are the respective means. Fig. f shows the probability of obtaining a parameter-2 angle at different multipliers of the standard deviation for data shown in fig. e. The probability obtained is used for scoring quadruplets. The third parameter is a torsion angle (fig. g) between the points j, mj, mi, i. mj is the midpoint between j+i, j-1. mi is the midpoint between i-1, i+1. Lines joining residues and the midpoints are shown in blue. A similar torsion angle involving residues j-1, i+1 as end points and midpoints between j, j-2 and i, i+2 is computed (not shown). Deviation of the torsion angle approximates the deviation of vertex i in fig. b with respect to vertex j due to rotation of the plane i-1, i, i+1 on the Z axis. Fig. h shows the distribution of torsion angles (binned at 5° intervals) obtained from DSSP output where c1 (-20.9) and c2 (-27.9) are the respective means for data from parallel and antiparallel β-strands. Fig. i shows the probability of obtaining a torsion angle at different multipliers of the standard deviation for the data in fig. h.

In this step, paired β-strands are generated to identify and represent sheet-forming β-strand ladders (including β-bends and β-bridges) which are paired stretches of consecutive residues. We start by defining and identifying the smallest unit of such a network of residues, namely a quadruplet, which is formed by four C_α _residues, linked by a pair of covalent bonds and a pair of hydrogen bonds (fig. 5a).

Since the covalent bonds that link a quadruplet of residues are easy to define confidently from their sequence and coordinates, and their hydrogen bonds are not always clear, we decided to use parameters that depend on covalently linked rather than hydrogen bonded residues neighboring the quadruplet (fig. 5). At most, a single covalently linked atom is required from outside the quadruplet at each of the four corners for calculation of quadruplet-scoring parameters. DSSP [8] output, obtained from the "Statset" described above, was used to identify parallel and anti-parallel quadruplets and to score the individual parameters that were considered. Only residues marked "E" in the DSSP "structure" column and having pairs under either or both "BP1" and "BP2" columns were used for generation of statistics. The neighboring atoms, if required by the parameter, were also required to be marked "E" under the column for "structure". We describe the three parameters used for scoring the quadruplets.

**The first parameter** C_α_-C_α _distance between paired residues (fig. 5a), is equivalent to the deviation of vertex i of the imaginary triangle i-1, i, i+1 from the vertex j of the triangle j+1, j, j-1 (fig. 5b). C_α_-C_α _distances from binned DSSP [8] data follow a normal distribution (fig. 5c). The distances for parallel and anti-parallel β-strands were computed. The distance data were fitted to the following equations for parallel and anti-parallel quadruplets respectively.





where ρ is probability of *x*, α is a constant, μ is mean and σ is standard deviation. GNUPLOT [40] was used to fit the data and obtain values for mean (μ) and standard deviation (σ).

Probability of obtaining a particular distance, for scoring quadruplets, is calculated by using the mean and standard deviation obtained above. Two distances are calculated for each quadruplet. For parallel quadruplets, both distances are scored individually using the mean and standard deviation from equation 4. For anti-parallel quadruplets, the larger distance is scored using μ_2 _(c3 in fig. 5c) and the smaller distance using μ_1 _(c2 in fig. 5c), and standard deviation obtained from equation 5 above. Two scores are used for each quadruplet (fig. 5a: distance between residues i, j and i+1, j-1).

**The second parameter** is an angle to determine the deviation of a residue pair with respect to the neighboring one. The angle between the line joining potential hydrogen-bonded residues and the line joining the previous and the next residues of one of the pairing residues was measured, for each of the pairing residues (fig. 5d). Angles were measured for parallel and anti-parallel β-strands. Mean (μ; c1 and c2 for parallel and anti-parallel β-strands respectively in fig. 5e) and standard deviation (σ) were calculated from the raw data. Times-sigma deviation was calculated for each data point (fig. 5f) and the probability of obtaining a value at each point was calculated and used for scoring quadruplets. Four angles are calculated and scored for each quadruplet (fig. 5d: angle between lines i, j and j-1, j+1; j, i and i+1, i-1; j-1, i+1 and i, i+2; i+1, j-1 and j, j-2). From this parameter, only half the total score is used to score the quadruplet.

**The third parameter **is the torsion angle between pairing residues calculated as an angle between planes joining the pairing residues and the midpoints between the neighboring residues (Fig. 5g: residue j; midpoint mj between residues j+1, j-1; midpoint mi between residues i-1, i+1; and residue i for the first torsion angle. Residue j-1; midpoint between residues j, j-2; midpoint between residues i, i+2; and residue i+1 for the second torsion angle.). Angles were measured for parallel and anti-parallel β-strands (fig. 5h) and 5a probability vs. times-sigma deviation curve (fig. 5i) was prepared similar to that of parameter 2 and used for scoring quadruplets. Two torsion angles are calculated and scored for each quadruplet.

Quadruplet parameters are scored based on the probability of obtaining individual Z-scores. Deviation of each parameter data from its respective mean value, obtained from DSSP, is divided by its respective standard deviation, obtained from DSSP, to obtain a Z-score (times-sigma value). Probability of obtaining a particular Z-score is used for scoring quadruplets.

As the probability values are very small and could be subject to floating point errors over multiple operations, a negative logarithm was used to convert them to positive numbers. Thus, lower numbers represent better scores. The total score of the quadruplet is obtained by adding the individual parameter scores. Equal weights (2 scores for C_α _distance, half of 4 scores for the second parameter angle and 2 scores for C_α _torsion angle) are used for the three parameters. Technical limitations of the computer's ability to work with numbers close to zero were carefully avoided by rejecting probabilities close to zero (less than six decimal places). The table of times-sigma and negative logarithm of the probabilities were kept for lookup during scoring of actual quadruplets found by DSSP [8] during determination of quadruplet scoring cutoffs, and by our algorithm during β-strand definition.

**Figure 6:**
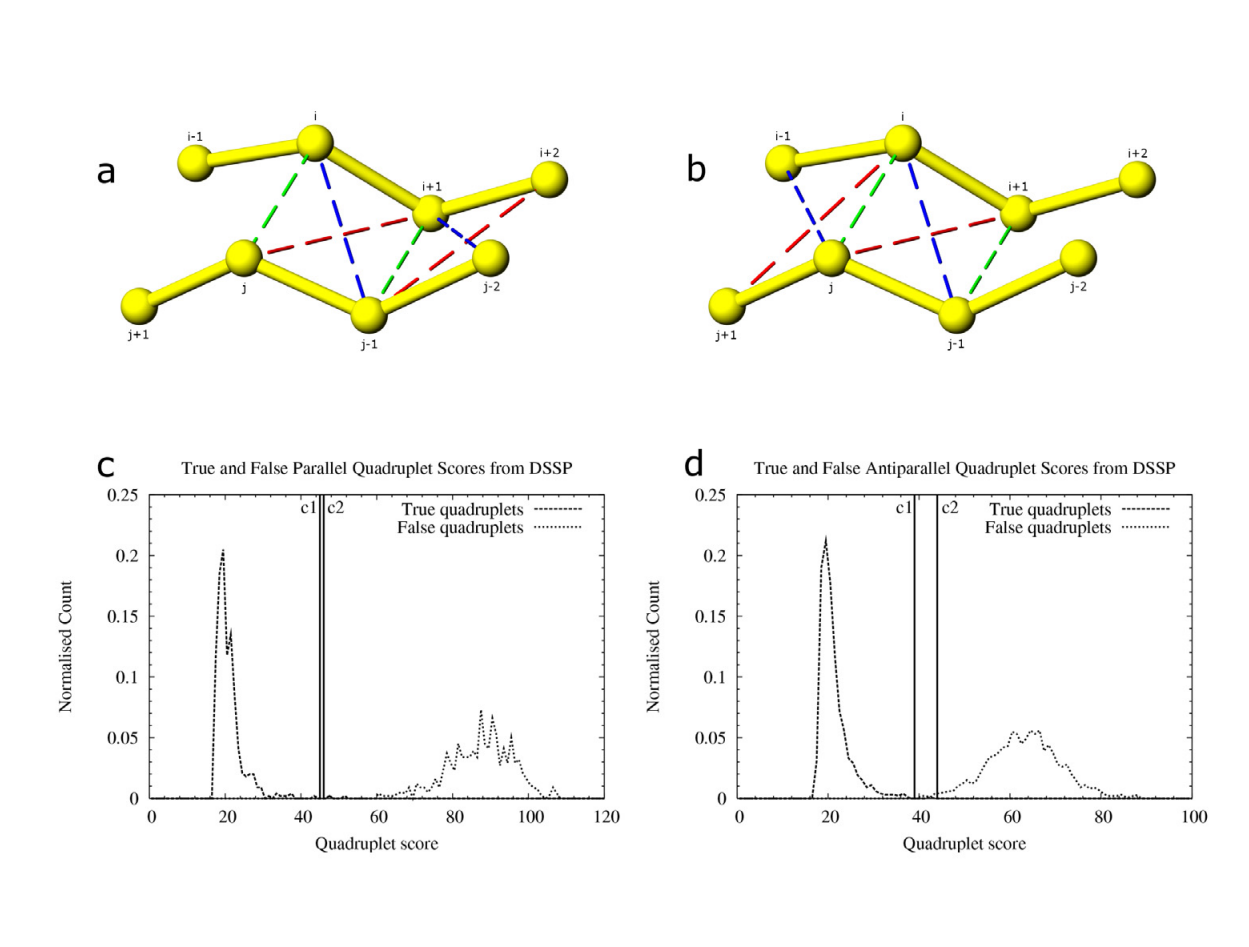
True and false quadruplets generated from DSSP-defined β-strands. 6a, 6b: Residues i, j and i+1, j-1 shown paired with green broken lines form the true quadruplet. For every such quadruplet four false quadruplets (shown with red and blue broken lines) are possible: j, i+1, i+2, j-1 and j-1, i, i+1, j-2 in fig. a; i, j-1, j, i-1 and i, i+1, j, j+1 in fig. b. These quadruplets were scored to find the difference in scores between true and false quadruplets. True and false scores for quadruplets generated from DSSP output for parallel β-strand coordinates (fig. c) and antiparallel β-strand coordinates (fig. d). Cutoff c1 (45) and c2 (46) in fig. c are the scores of the best false quadruplet and the worst correct quadruplet respectively for parallel β-strand data. Cutoff c1 (39) and c2 (44) are the scores of the best false quadruplet and worst correct quadruplet respectively from antiparallel β-strand data. Cutoffs c1 and c2 are used as cutoffs to differentiate between grade 1 and grade 2 quadruplets in our algorithm.

Sets of true and false quadruplets were generated from DSSP [8] output (figs. 6a, 6b). A true quadruplet was created using two pairs of consecutive residues with a link in the "BP1" or "BP2" column of DSSP output. For every true quadruplet that was created, four false quadruplets were generated as shown in fig. 6 by considering alternative quadruplets that these residues could potentially form with neighboring residues. Each quadruplet was scored using all three parameters (fig. 5) as described above. Scores for true and false quadruplets were analyzed (figs. 6c, 6d).

Our algorithm uses quadruplets formed from all available residues with the restriction that both covalently linked residues do not exist in any strict-helix SSET and allows a maximum overlap of one residue with a strict-helix SSET. Pairing residues are limited by C_α_-C_α _distance (7.5 Å; fig. 5c), before scoring the other two parameters to improve computation speed. Since quadruplet-based β-sheet definition is one of the most computationally intensive steps of the algorithm, we critically examined distance scores to reject quadruplets that will not be part of a β-sheet. The quadruplets are scored similar to quadruplets obtained from DSSP [8], as described above. Based on the final score and cutoffs determined from the scores of true and false quadruplets generated from DSSP, quadruplets are placed in one of three groups. The *grade 1 *quadruplets are those with scores better than the best false quadruplets located from DSSP in the previous step (quadruplets scoring less than c1 in fig. 6c and 6d). The *grade 2 *quadruplets are those scoring in between the best false DSSP quadruplets and worst true DSSP quadruplets (quadruplets scoring between c1 and c2 in figs. 6c and 6d). Lastly, the *grade 3 *quadruplets are those, which passed cutoffs of distance and have non-zero probabilities for each of the three parameters (fig. 5). These three grades of quadruplets are sorted based on their scores and used as seeding and extending units for all paired β-strands.

### Step 4: Calculation of cutoffs to disallow neighboring quadruplets with wrong residue pairings

Use of relaxed criteria such as those described in the previous section to determine the quality of quadruplets can lead to errors in some cases. For example, it is possible to obtain a low scoring grade 1 or a grade 2 quadruplet in cases where an extended region comes close to a β-sheet, leading to quadruplets connecting the β-sheet to the extended region. Depending on the local structure, these quadruplets may score better than those of the β-sheet in the surrounding region. While it is possible that one or a couple of good hydrogen bonds might actually exist in such a situation between the β-sheet and the extended region, this region will now get incorrectly assigned to the sheet. In such a case, the secondary structure obtained in a residue-correct manner will not be beneficial for the purpose of approximating β-sheets as a set of interacting linear elements.

**Figure 7:**
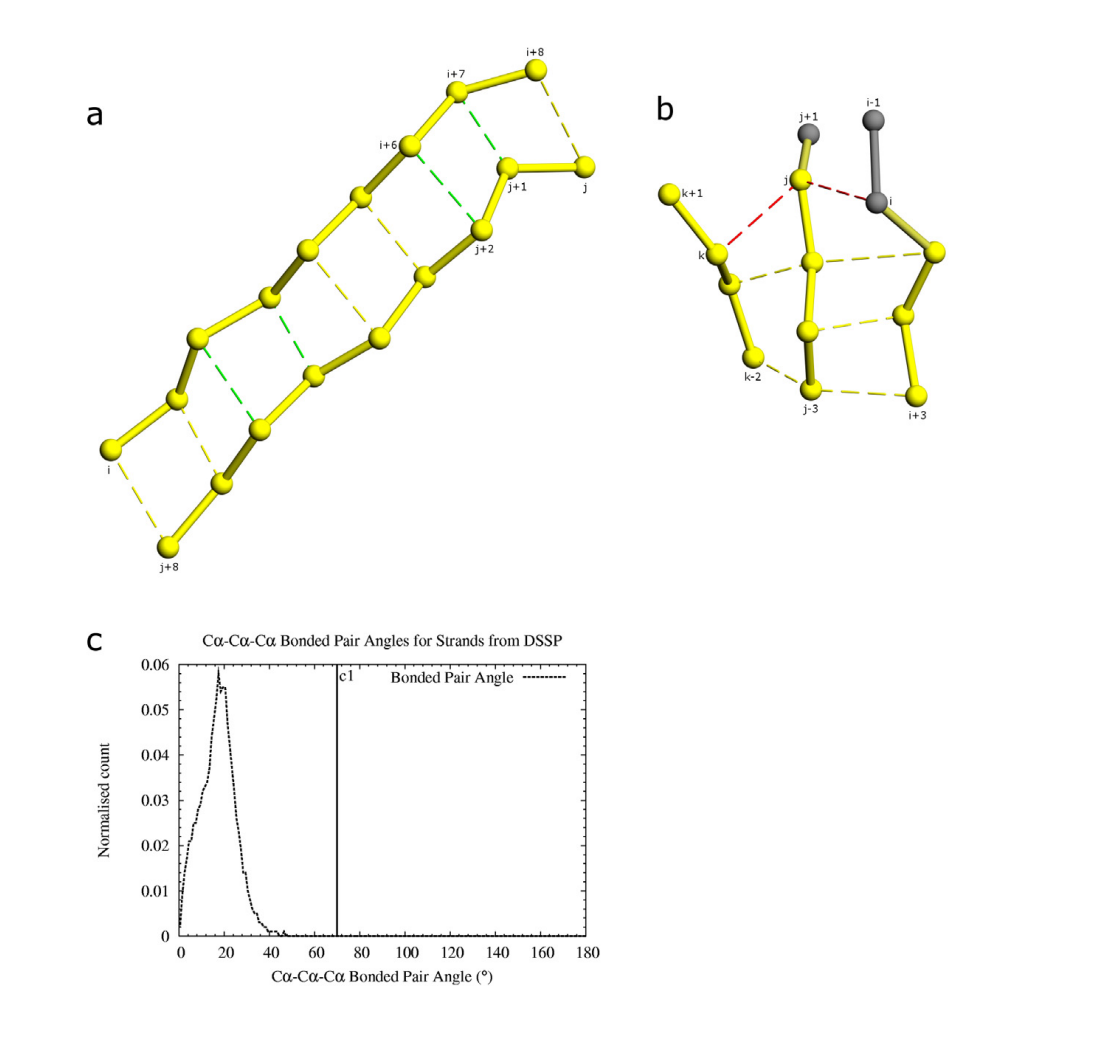
Initiation and extension of ladders of paired residues using quadruplets. 7a: Ladders of paired residues are initiated and extended using quadruplets. The initiation quadruplets i+2, i+3, j+5, j+6 and i+6, i+7, j+1, j+2 are shown with green pairing. Quadruplets are attached on either side to extend the arms of the ladder. Addition of quadruplet i+4, i+5, j+3, j+4 joins the two ladders i, i+4, j+4, j+8 and i+5, i+8, j, j+3 to form the complete unit. Depending on the position of the best quadruplets any number of quadruplets might be responsible for seeding a ladder. Smaller ladder fragments get joined by worse scoring quadruplets. 7b: Residue pairing angle between residues on three β-strands (residue i+1, j-1, k-1 in fig. c) from DSSP output. Cutoff c1 (70°) is close to the largest angle observed. This was used to check new residue pairings formed while adding quadruplets. 7c: Checks performed during quadruplet addition and ladder extension. Quadruplet k, k-1, j-1, j and i, i+i, j-1, j share the common residues j-1 and j. Pairing and angle between pairs are checked for residues j-1 and j when worse scoring quadruplets are added. Quadruplet k, k-1, j-1, j scores better than i, i+1, j-1, j. While adding i, i+1, j-1, j it was found that the angle i, j, k fails the cutoff of 70° (fig. b). Quadruplet i, i+1, j-1, j is not added. Insertion of bulge residues is handled during joining of quadruplets. Quadruplets i+1, i+2, j-2, j-1 and i+2, i+3, j-3, j-2 share the common residues i+2, j-2. The quadruplets are simply added end to end. However, quadruplets j, j-1, k-1, k and j-2, j-3, k-2, k-1 (pairing between j-2, k-1 not shown) share only a single residue k-1. As j-2, j-3, k-2, k-1 scores worse than j, j-1, k-1, k, the pairing between j-1, k-1 is retained and residue j-2 becomes a bulge with respect to residue k-1.

A rule based on multi-strand β-sheet geometry was implemented to determine correct quadruplet extension and pairing in later steps. True quadruplets generated in the previous step from DSSP [8] output files (figs. 6a, 6b) were studied with respect to the bending angle of neighboring quadruplets having a common pair of covalently bonded residues. Angles were calculated for each of the common pair of residues with its pairs from both quadruplets (figs. 7a, 7b). An angle of 70° was chosen (c1 in fig. 7b) as the cutoff to determine if neighboring quadruplets are part of the same β-sheet. Both residues of the common covalent quadruplet edge have to pass the cutoff for both neighboring quadruplets to exist in a β-sheet. This ensures that no residue in any β-sheet has an angle >70° with its pairs and solves the problem of incorrectly assigning an isolated extended region that happens to be in the vicinity of a sheet as being a part of that sheet.

### Step 5: Initiation of β-ladders (paired β-strands)

A quadruplet of residues can potentially be extended by joining neighboring quadruplets in the direction of hydrogen bonds and/or in the direction of covalent bonds, to form a β-sheet. However, extension in the direction of hydrogen bonds poses some problems in the case of bulges. Therefore, we decided to extend quadruplets only in the direction of the covalent bonds in order to obtain β-strand ladders (fig. 7c). The quadruplet extension process leads to the formation of ladders that are a sequence of paired residues. Thus, all residues on one arm of the ladder are paired with neighboring residues on the other arm of the ladder (fig. 7a), except for bulges (fig. 7b). Presence of a bulge is considered during ladder generation, and it is possible to have isolated residues on one arm of the ladder that do not have a pair. Such a bulge residue is incorporated into a ladder at this step.

Generation of paired β-strands is initiated using quadruplets from the sorted list of grade 1 quadruplets obtained in step 3. Use of only grade 1 quadruplets for seeding ensures that the core region of paired β-strands is less prone to errors. Each grade 1 quadruplet, starting with the best scoring one, is checked for its ability to either join a previously selected better scoring quadruplet or initiate a new ladder in this single pass method. In case of a conflict with a previously chosen quadruplet, the current worse scoring quadruplet is rejected. In every step irrespective of whether the current quadruplet is chosen or rejected, all quadruplets in the grade 1, 2 and 3 lists having scores worse than the current quadruplet and which can possibly conflict with the current quadruplet based on its constituent residues and pairing are rejected. This ensures that conflicting quadruplets do not initiate ladders on their own. Attempts are made to join quadruplets end to end in one of two ways. A check is first made if both edge residues (fig. 7a) of one quadruplet are the same as both edge residues of the neighboring quadruplet. This allows two consecutive quadruplets to be joined end to end to extend the ladder by a pair of residues. Upon failure to join a quadruplet by this method, the second method is applied. If only one corner of a quadruplet has the same residue as a corner of the other quadruplet, and the pairs to that corner residue in the different quadruplets are consecutive, the quadruplets are joined end to end to extend the ladder by one pair of residues. The pair for the common corner in the new quadruplet is designated as a bulge with respect to the common corner residue. This method of generating bulges also ensures that bulges arise from the worse scoring quadruplet. We have found such an assignment to be correct by manual inspection. In case no suitable existing quadruplet is located at either edge of any preformed ladder, the current quadruplet is designated as a ladder by itself and can be extended by quadruplets scoring lower than it.

Joining of quadruplets is attempted for both edges of a quadruplet. Thus, a quadruplet can potentially join with the edge quadruplets from two different ladders, with its own two edges. This joins the two existing ladders to form a single longer ladder of quadruplets. These ladders of paired residues are checked for errors before further processing. It is possible to obtain quadruplets with both residues on its diagonal as bulges. All such quadruplets are removed after breaking the links at their edges with neighboring quadruplets. Quadruplets are selectively removed even if a single common residue belonging to a pair of neighboring quadruplets fails to pass the cutoff angle for wrong residue pairing as described in step 4 (fig. 7a). Cases where residues have two and three pairing residues are treated differently. A single residue, pairing with more than three other residues was not observed, thus showing that quadruplet parameters are able to distinguish between gross errors in geometry. A residue having only two pairs indicates two neighboring quadruplets. If the residues involved fail to pass the cutoff scores for the angle, the worse scoring quadruplet is rejected. A residue pairing with three other residues indicates three neighboring quadruplets having at least one common residue. It is possible for a set of three residues at this location to pass cutoff scores for angle, in real structures, so we choose and eliminate the wrong quadruplet at this location. Two distinct cases are handled. A quadruplet is removed if it does not pass the angle cutoff with another neighboring quadruplet. If two quadruplets do not pass cutoff within themselves, but both pass cutoffs with a third quadruplet, we keep only one of the first two. This is chosen based on the length of the ladder of paired residues which the quadruplet takes part in. The quadruplet from the longer ladder is retained while the other one is rejected, consistently with the goal of obtaining longer elements. All isolated single quadruplets, which are formed by residues between two strict-helix SSETs, are removed, as they cannot extend a β-strand in any direction.

### Step 6: Removal of short helices between consecutive β-strands

In our program, β-turns tend to show up as five residue helices. This has also been observed in other algorithms that define secondary structure on the basis of C_α _coordinates [7,15,21]. Since we prefer fewer long elements instead of many short connected elements, wherever possible, β-turn regions are shown as part of the β-strand instead of short isolated helices. A step was added to remove all small helix SSETs at β-hairpin regions. We define a helix as being a minimum of five residues, as this could suitably represent a single turn of a helix. Moreover, for removal at this step, a helix is required to have at least five consecutive residues that are not part of any β-ladders formed in the previous step. We consider a maximum overlap of two residues between elements, so all helices of eight residues or longer always pass this criterion. Thus for this step, all helices up to seven residues long are considered for removal. Only helices inside β-hairpin bends as detected by C_α _pairing between the β-strands are removed.

### Step 7: Extension of β-ladders

Quadruplets with grade two scores are used to extend ladders formed from at least two grade 1 quadruplets. All ladders therefore have a core region of grade 1 quadruplets, which are then extended to the regions that are more distorted. This approximates stretches of paired residues with definite hydrogen bonding extended by residues where strict hydrogen bonds may be absent, and prevents defining entirely incorrect β-strands. Extension is very similar to the initiation step using grade 1 quadruplets, except that more checks are put in place when each quadruplet is added, to prevent unreasonably distorted regions from being joined. Our algorithm attempts to approximate elements as linear vectors at every step of the process. We incorporated steps to geometrically define the end of a ladder of paired residues to prevent generating ladders that span more than a single linear element. Quadruplets are not added if these criteria are violated. As grade 2 quadruplets are formed using less stringent criteria than the grade 1 quadruplets, a conservative approach in their use prevents delineation of highly distorted regions as β-strands. Extension is attempted on a list of ladders sorted by length. This ensures that longer elements have a better chance of ending due to geometric criteria rather than due to an extension quadruplet already being allocated to a shorter ladder. A pair of residues are added to the ladder only if neither of the new residues already have two other residue pairs each. Thus, ladder extension depends both on ladder length as well as on quadruplet grade. Extension of a ladder is also terminated if this causes residue overlap with residues already participating in a strict-helix SSET. Only residues that are a part of a loose-strand SSET are used in the extension step. Extension is also terminated upon reaching a β-hairpin bend. No residues are added if the pair about to be added does not fall within the distance cutoffs of 3.5 Å to 7.5 Å (fig. 5a). The ladder is terminated, if any of the residues about to be added have an i-3, i C_α_-C_α _distance less than 8.1 Å (eq 1, fig. 4c) with a residue already in the same ladder. This ensures that we do not extend a ladder over a β-bend.

Grade 3 quadruplets are used next, to extend only single grade 1 quadruplets that have not been extended by any of the above methods. As grade three quadruplets are the worst scoring quadruplets that are used, two important constraints are utilized to prevent spurious extensions of lone grade 1 quadruplets. Firstly, we decided to use a criterion of exactly three consecutively paired residues to denote paired β-strands that arose from single grade 1 quadruplets. This ensures that only a single unreliable grade 3 quadruplet is used for extension of any particular β-ladder. Upon inspection of structures, it was observed in that keeping a minimum of two consecutively paired residues, namely a single grade 1 quadruplet, to delineate β-strands, was too relaxed. This constraint alone was able to eliminate most cases of chance interaction between pairs of residues. However, in highly distorted regions that are too twisted to represent linear elements, more than a pair of consecutive residues seem to form isolated ladders. We decided not to include them as well. So, secondly, only grade 1 quadruplets sharing common residues with an existing β-ladder, formed by previous initiation and extension steps, are extended by a single grade 3 quadruplet, such that it maintains residue pairing with the longer β-ladder. These two constraints together ensure that we retain short, three residue, β-strands at edges of existing β-sheets even if they are slightly distorted, but at no other region unless they are initiated by a pair of consecutive grade 1 quadruplets.

At this stage, due to the possibility of bulges at the overlapping edges of consecutive β-ladders, we might obtain consecutive ladders of residue pairs whose one arm is separated from the other by a single residue. These ladders are joined with a single bulge residue if consecutive pairing arms on the other side share a common residue and restrictions imposed during ladder extension are not violated. Our method of ladder generation also makes it possible to obtain overlapping ladders such that one arm of one ladder overlaps with the arm of a different ladder. Due to the complicated nature of β-sheets, it is also possible to not have residue pairing between neighboring β-strands in the middle of a β-sheet. Dealing with multiple fragments of overlapping ladder-arms prevents the ability to distinguish between true edges of β-strands. Thus, we join all ladder arms that are formed from consecutive stretches of residues or with common residues between them. These joined ladder arms are used to denote β-strands. The residue pairing information generated during ladder formation is retained to generate pairing information between the newly formed β-strands. Consistent with our minimum requirement of three pairing-residues for ladders (described above), β-strands are considered as part of the same β-sheet if at least three residues are paired.

### Step 8: Generating and breaking α-helices to form linear elements

Helix SSETs representing α, π and 3_10_-helices, defined in the above steps, are based on relaxed criteria to avoid missing out residues that could potentially be part of a helical element. Although a rudimentary form of element edge delineation is obtained by the use of C_α _distance and torsion angles during delineation of probable helical elements in step 2, these methods are capable of detecting only drastic changes in the helix axis. Our relaxed criteria of helix definition designed to include π and 3_10 _helices, also allows curved, kinked and bent helices [44] to be included as being a part of a single element. In order to approximate these elements by vectors suitable for motif search, we decided to split these helices into linear elements while considering overlap between them. Some helix SSETs are found to overlap with parts of ladders generated and extended in the previous step. As the residues that overlap are not part of strict helix SSETs we prefer a state of 'β-strand' to 'α-helix' for the residues. Helix SSETs having β-strand overlaps are checked and shortened so as not to have more than two residue overlap, and only at the edges, with any residue that is part of a β-strand. As it is possible for a residue to form only two hydrogen bonds, we simulate this constraint by rejecting from the edge of the helix any residues that pair with more than one other residue in the formation of β-strands in the above step. The helices are again checked for the presence of at least five residues before being accepted for processing by this step.

Manual judgment of the results at this point indicated that our program's delineation of helices were acceptable in terms of residue coverage. Presence of bent helices was noticed in the checkset (described previously) and we decided to split them into linear elements without loss of constituent residues. We used helices defined by our program for calculation of parameters for breaking helices. Since DSSP [8] does not distinguish between consecutive helices, it was not possible to derive parameter-based cutoffs from DSSP helix definition. Using data from helices defined by our program, enabled us to implement a system that could properly handle loosely defined helices and not to break them more frequently than needed. The results were visually inspected for errors.

**Figure 8:**
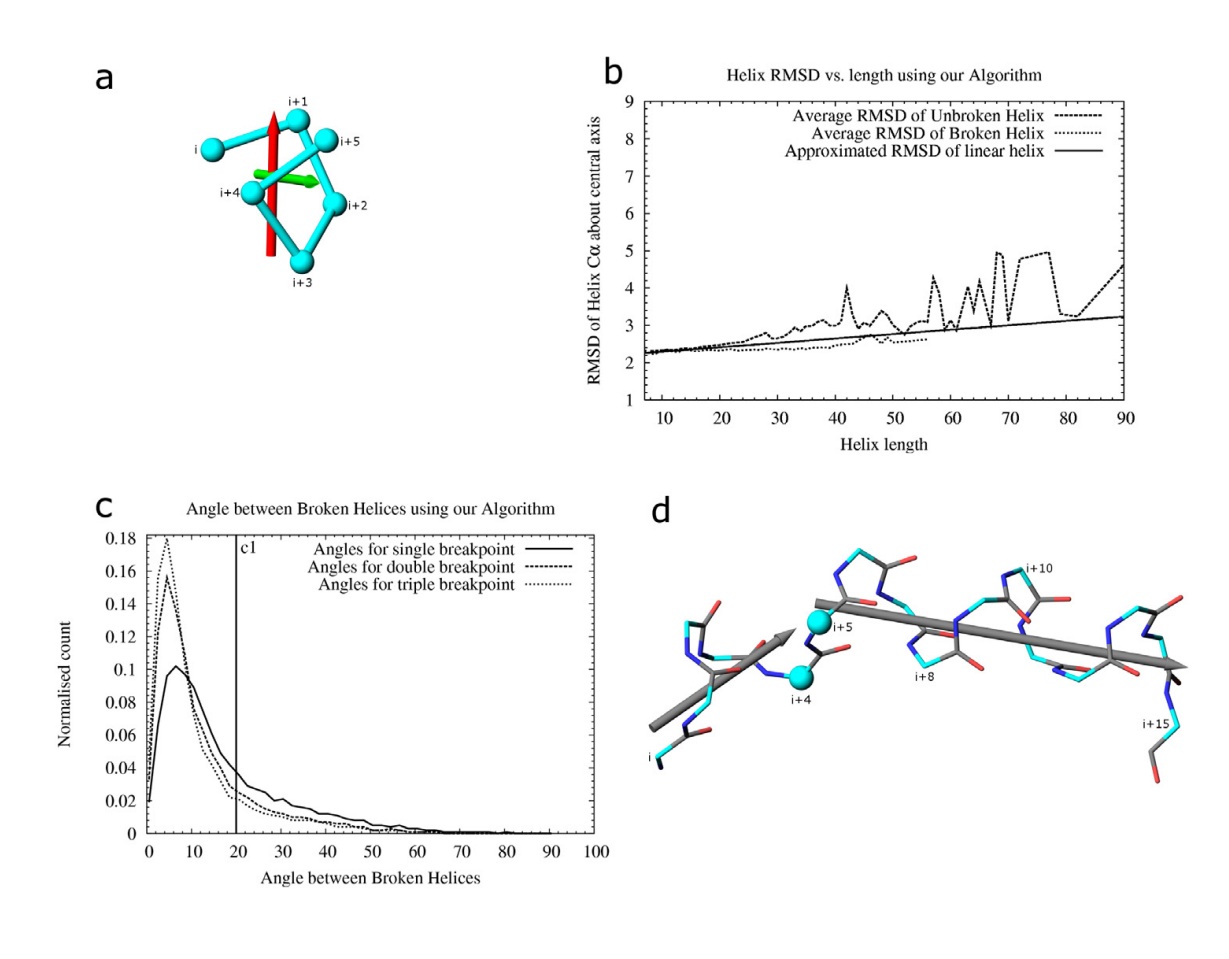
Helix endpoints redefined based on RMSD and angle between their axial vectors. 8a: Vectors representing a short opened up helix by two different methods. The red arrow shows the axis obtained by using the largest spread of the C_α _atoms (vector corresponding to the largest eigenvalue). The green arrow shows the rotational axis obtained when the helix that is shifted by one residue, is aligned to the original helix. The first method is unsuitable for representing this helix and does not work for π and short helices. Our algorithm uses the rotational-fit method (described below, 8b) for all helices. RMSD of residues are calculated over this vector. Angles between vectors, calculated from residues of consecutive helices, are used to determine whether to break them so as to appropriately define the helices as linear elements. 8b: Helix RMSD data calculated using the rotational fit vector. Average RMSD of unbroken helices from our algorithm varies widely. The helices were broken multiple times and the angle of break was analyzed (data not shown). The mode of the angle of break (22°) for long (>15 residue) helices was used to determine the break point of consecutive helices. Helices that break at >22° were chosen for the dataset for calculation of RMSD and angle of break (fig. c). Average RMSD of broken helices is shown in this figure. A line was fitted using "gnuplot" [40] to approximate the RMSD of broken helices. A Z-score of 2.5 is used to limit breaking helices that deviate less than 2.5 times sigma around the approximated RMSD value for broken helices at a particular helix length. 8c: Angle of helix break calculated from dataset of helices used in fig. b. Data were collected from helices broken once, twice and thrice. The normalized data are shown. Helices that show an angle greater than c1 (20°) between broken parts are split. 8d: Helix split by our algorithm. All possibilities of broken pieces are assessed with respect to the RMSD of the pieces and angle of break. Helices i, i+5 and i+4, i+15 are finally chosen as correctly broken. Helices i, i+8 and i+15; and i, i+11 and i+10, i+15 are also possibilities that are analyzed but not chosen as the optimum break. Residues i+4, i+5 are shared by the two helix pieces (C_α _shown as spheres).

The helix breaking method relies on an analysis of the RMSD of helix residues around the helix axis. Two different methods were used to generate the helix axis. Only one of the methods was finally adopted (fig. 8a). We explain both methods, as they are equally suitable for long (>7 residue) α-helices. However, the method chosen for our algorithm works for helices defined by us which encompass α, π and 3_10 _helices as distinct or part of the same element and also short (<8 residue) α-helices. The first method calculates the principal moment of the helix residues and used the eigenvector corresponding to the largest eigenvalue as the helix axis. This method thus depends only on the spread of the residues in space and does not take into account the linear connectivity of the helix residues. Errors in helix axis assignment were observed for π-helices and short α-helices. Due to the spread of residues being more on the diametrical plane of these helices, the axis found using the eigenvector method lay closer to the plane of the diameter instead of being normal to it. This method gave good results for longer helices. However, as we considered short α, π and other opened up helices for breaking and final definition, this method was not used.

Based on the observations above, a rotational fit method was used to determine helix axis [22,45] (fig. 8a). This involves shifting the helix by one residue and aligning it with the original helix. The rotation axis corresponds to the helix axis. This was found to precisely determine the helix axis and was not dependent upon the length or under-winding of the helix. The axis determined by this method and that determined by the eigenvector method correlate very well for helices greater than 8 residues (data not shown).

Our program was run on the "statset", described previously, and all helices were extracted for study. The RMSD of helix residues from the helix axis were calculated and analyzed (fig. 8b). All helices were processed using the helix-breaking method described below. Each helix was split in three ways such that case 1 gave rise to 2 pieces, case 2 gave rise to 3 pieces and case 3 gave rise to 4 pieces from the original unbroken helix. The mode of breaking angles was calculated for every helix length (data not shown). The highest mode (22°) obtained was used in our breaking method to obtain a set of all helices that broke at least once at an angle greater than the mode. Broken helices that arose from this set were used to calculate the RMSD of broken helices around the rotational fit axis (fig. 8a). The standard deviation was calculated for every helix length and the results showing dependency of the standard deviation on the length were fitted to a straight line (data not shown).

The average RMSD and average standard deviation calculated above were used to obtain broken helices (as described below) that were analyzed manually. Our algorithm does not break a helix showing a slight curvature in its structure, or containing a few distorted residues. For bent helices, we decided to use two cutoffs to determine breakpoints. Flexibility is represented by a Z-score representing the allowed deviation as multiples of the standard deviation of helix residues (as calculated above). A sharp bend in the helix axis is measured by the angle between the axes of two neighboring helices as calculated by the rotational fit method (described above). The broken helices observed were manually inspected to determine the optimum Z-score and the break angle (fig. 8c).

A breaking method for helices was developed in order to determine the correct cutoffs for Z-score and angle of break. The method considers every possible breakpoint in a helix and attempts to choose the optimal result. We define broken helices as single elements only if the piece with the highest RMSD is still acceptable (fig. 8d). Multiple breaks are considered with no overlaps and with overlaps of up to two residues at every position of the helix. The minimum helix length allowed is five residues. Broken helices are considered starting from the shortest helix length (5 residues), with maximum overlap (2 residues), to the longest possible helix (the unbroken helix). The helix breaking process analyses the RMSD and angle of break for every combination of helical fragments (instance) derived from the unbroken helix. Thus, every "instance" is the set of broken helical fragments produced during the iterative breaking process. Every possible case of broken helices that can be formed by an unbroken helix is considered and a single helix is broken into as many long fragments as is optimal for correct representation as linear elements. RMSD along the helix axis, computed by the rotational fit method described above, is used to determine overall suitability of broken helical pieces. For every unbroken helix, RMSD of every possible broken helical fragment is considered. For all "instances" of broken helices, the fragment with the highest RMSD is located. The "instance" where the highest RMSD is minimum is considered the optimum break. Thus, we aimed to minimize, over all possibilities in which a single helix may be broken, the maximum RMSD of the broken helices generated by a single "instance". A Z-score cutoff is used to determine if a helix or helix-fragment needs to be broken. We tried out different values of Z-scores in an attempt to find the optimum value. The results for every set of broken helices corresponding to different Z-scores were manually inspected to disallow breaking helices that looked like a single element. Consecutive fragments are treated as a single helix if the angle between their axes is less than the cutoff angle, even if the resulting helix has a Z-score that fails cutoffs. A Z-score cutoff of 2.5 and an angle cutoff of 20° (fig. 8c) were finally chosen. Results were judged manually for correctness. Linear helices, according to our algorithm, thus never fail the axial bending angle of 20° and are not broken. This is close to the 25° angle obtained by Kundrot and Richards [15]. Curved helices are broken only if they are long and the axial bending angle is above 20°. Bent and kinked helices are already broken in step 2 (by i, i+3 C_α _distance and i, i+1, i+2, i+3 C_α _torsion cutoffs; fig. 4c, 4d) as they have a sharp angle and never need to be broken by this step.

This step of the calculation is computationally intensive due to the large number of possibilities that are considered. To prevent the algorithm from taking abnormally long to complete in special cases, we avoid checking for breaks in single helices that are longer than 50 residues.

### Step 9: Breaking β-strands to generate linear elements

Bent β-strands are split to obtain linear elements using both geometric criteria (C_α _distance and angle) and by using neighbor pairings for constituent residues. As β-strands have a natural tendency to curve, we take care not to break short gently curved β-strands, nor break them at bulges. Sharp bends in β-strands, gently curved but long β-strands, which cannot be optimally represented by a single linear vector, and β-strands which do not have at least two residues shared by two different β-sheets are considered for breaks. β-strands are broken using geometric criteria after checking for the possibility of a bulge being located near the potential breakpoint. We prefer to retain large regions of connected β-strands rather than split them into isolated pieces, and therefore place more importance on residue pairings than on geometric criteria of individual β-strands. Restricting the minimum length of a β-strand to three residues (as described above) makes β-strand breaking a sensitive operation, as it is possible to lose small β-strands completely if breaks are located either within the small β-strands themselves or on the β-strand pairing with the small β-strand. Thus, we try to retain residues in short β-strands if they are linked to part of a larger β-sheet. However, it is more likely for short β-strands to arise due to chance proximity of extended regions. These short β-strands may hinder correct representation of the entire β-sheet. Although our program does not aim to perfectly describe β-sheets, we do try to detect and remove short β-strands that are either not well connected to a larger β-sheet or located close to breaks in neighboring β-strands of the β-sheet. The β-strand breaking methods are designed not to depend on the length of the β-strands being broken, however, they do depend on the order in which they are applied on the original β-strand and its paired neighbors. β-Strands are broken in four steps, where each step works on the complete set of β-strands obtained after applying the previous breaking method. Bulges are taken into consideration at every step.

β-hairpin regions of β-strands are broken first, and these breaks are permanent. The use of quadruplets to generate β-ladders and joining ladder-arms to obtain paired β-strands does not allow demarcation of β-hairpin regions perfectly. Also, use of β-hairpin residues to form part of β-ladders in previous steps is not consistent with our aim of using a quadruplet to represent pairs of hydrogen bonded residues, since the last pair of residues of a hairpin bend may be consecutive and thus covalently bonded. Further, residues of hairpin bends, even when separated by a single residue score low when included in quadruplets. We identify all β-hairpins using residue pairing. This enables β-hairpin identification even if the last pair of paired residues leading to the hairpin is separated by up to three residues. This method allows detection of a β-hairpin correctly if the β-hairpin residues are paired with neighboring β-strands but not between themselves. All such cases of β-hairpins are resolved by placing the residues in one of the two β-strands based on residue pairing of consecutive residues. In case of an odd number of residues being in the β-hairpin, the middle residue is left out of the β-strands on either side of the β-hairpin.

**Figure 9:**
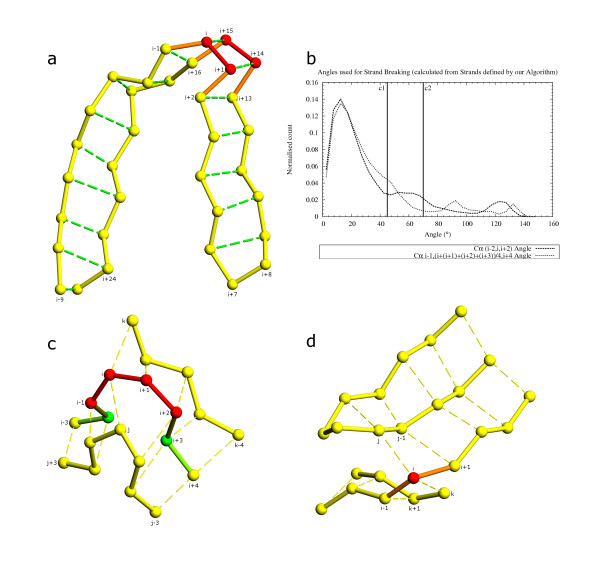
β-strands redefined to obtain linear elements using different methods. 9a: Strands broken based on i, i+3 C_α _distance (fig. 4a). Distance between i-1, i+2 residues and i+13, i+16 residues fail the cutoff distance of 8.1 Å. The residues i, i+1 (shown in red) are shared by β-strands i-9, i+1 and i, 1+7. Residues i+15, i+14 (shown in red) are shared by the β-strands i+24, i+14 and i+15, i+8. 9b: Angles for β-strand breaking while accounting for bulges. Angles were calculated from all β-strands defined by our algorithm before the β-strand-breaking step. The angle between i-2, i, i+2 C_α _atoms is used to determine if the β-strand is bent. An average pseudo-point (pp) was generated from the j, j+1, j+2, j+3 atoms and the angle between j-1, pp, j+4 was found. β-strands were broken when i-2, i, i+2 angle was greater than c1 (45°) and j-1, pp, j+4 angle was greater than c2 (70°). j = i-1 showed the best correlation between the two angles (data not shown). 9c: Strand breaking using pseudo-point to find distorted regions. Residues i-1, i, i+1, i+2 (shown in red) are used to generate an average point. Angle between i-2, the average point and i+3 locates a distorted region if the cutoff angle of 70° fails. The β-strand is broken if the i-2, i, i+2 angle also fails at the same location. The β-strand i-3, i+4 is split to generate two β-strands i-3, i+1 and i, i+4. 9d: Strand breaking using pairing information between neighboring β-strands. Residue i (shown in red) is paired to residue j on one side and to residue k on the other. Residue i+1 is paired to residue j-1 however residue i-1 is not paired to β-strand j. Also, residue i-1 is paired to residue k+1 but residue i+1 is not paired to β-strand k. Lack of a pair of common residues pairing between β-strand j and k splits the sheet, with residue i shared between both sheets.

Geometric breaks of β-strands using i, i+3 C_α_-C_α _distance are considered (fig. 9a). These account for the presence of bulges and are not marked as permanent breakpoints. Depending on pairing of and breakpoints in neighboring β-strands, the breakpoint may be disregarded in later steps. If the i, i+3 C_α _distance is less than the cutoff of 8.1 Å (c1 in fig. 4c) a break is inserted. Presence of a bulge, originating from β-ladder initiation and extension steps above, within any of the residues at i, i+1, i+2, i+3 positions, prevents a break. The residues at positions i+1 and i+2 are treated as overlapping residues and included in both β-strands. Although breaks generated by this method indicate structurally bent regions, they are discarded upon absence of other potential breakpoints in the vicinity, at later steps. Breaks are not initiated if they lead to less than three-residue long β-strands.

Geometric breaks of β-strands are also considered based on i-1, i, i+2 C_α _angle. The i ± 2 C_α _angle was observed to be a reliable indicator of bent β-strands in non-bulge regions. This break, as in the previous case, is treated as a potential break point only. A permanent break is initiated only if no bulges are observed in the β-strand region. All β-strands defined by our program were extracted from PDB [20] chains in the "statset" and the i-2, i, i+2 angles were calculated (fig. 9b). An angle of 45° (c1 in fig. 9b) was used as cutoff to determine a breakpoint. This was found to be too restrictive in that regions with only a single distorted residue could get broken. A method was implemented to locate β-strand distortions in the surrounding region using a less restrictive criterion. Data for finding cutoffs for bulge detection was found by processing the "statset" with our program. All β-strands defined by our program were extracted. A pseudo point was created for each set of four consecutive residues belonging to the same β-strand using residues i-1, [i, i+1, i+2, i+3], i+4 (fig. 9c). The angle between the lines joining the pseudo-point to the residues before and after the four residues used for the pseudo-point was calculated for all residues of the β-strands (fig. 9b). An angle cutoff of 70° (c2 in fig. 9b) was chosen to allow gradual bends in the structure. A break is allowed only if the i-2, i, i+2 C_α _angle and the pseudo-point angle both fail for a given residue i. Breaks are not initiated if they lead to β-strands less than three residues long.

The final method used for β-strand breaking considers residue pairing between β-strands. Using residue pairing for detecting the end positions of β-strands allows splitting a β-sheet correctly based on the whole structure and not on minor distortions in geometry. Breaks by this method are permanent and are used as deciding factors in splitting an entire β-sheet into smaller pieces. Although our program does not aim to produce a perfect definition of all β-sheets, with respect to their boundaries as appropriate for correct domain definition, we do verify the SSE pairings to include them as part of the correct β-sheet. Due to the way in which ladders of residue pairs (described previously) were generated and joined, it is possible for two ladder arms to be formed from consecutive residues or be with a maximum of only one residue common between them, with the residues on the other arms having no connectivity (fig. 9d). A minimum of two residues overlap is required for neighboring β-strands to be part of the same β-sheet. Thus, a break is initiated if residues of a β-strand pairing with one of its neighbors lose contact with it for more than a single residue, unless the other pairing β-strand continues to maintain residue pairing on the other side. Only one common residue is allowed at the break, thus allowing a single residue to pair with both the neighbors, which are placed in different β-sheets. A β-strand is not considered for breaks if there are at least two residues pairing with both neighboring β-strands. Flexibility in allowing lost contact for a single residue ensures that bulges are retained.

### Step 10: Generation of helices from residues not in any element

In previous steps, residues not part of a strict-helix SSET were considered for generation of ladders of residue pairs and β-strands. Some of these residues do not finally participate in any β-strand formation. These residues, not part of α-helices or β-strands, can potentially contribute to formation of π and 3_10 _helices, or may be distorted while still showing helical tendency. Overlap with previously defined α-helices and β-sheets were considered for this step.

All loose-helix SSETs having no residue in a previously defined α-helix or a β-strand are considered at this step. To loosen the criteria and to allow over and under-wound helices to be detected, any presence of a strict helix-forming residue, other than in the last three residues of the previously defined element, leads to rejection of the template from consideration, as these have already been considered for helices in above steps. The templates are checked for a maximum overlap of two residues with β-strand elements and shortened at the edges if required. The templates are also checked so that no residue overlapping with a β-strand at the edge, pairs with more than one β-strand residue. Finally, helix templates occurring at β-hairpins are removed. Any helix template that overlaps with β-strands on both edges and has less than five non-overlapping helix residues is rejected. All remaining loose-helix SSETs are included in our final helix definition.

### Step 11: Assignment of β-strands to β-sheets

As described previously, we consider a minimum of two pairs of residues to determine linked β-strands which themselves are at least 3 residues long. Consecutive residues of β-hairpins are considered linked for this purpose, even though they do not actually form a hydrogen bond. Sheets are assigned for each group of β-strands that can be traversed by a consecutive pair of linked residues. Breaks in β-strands located in previous steps are taken into consideration for this step. Breaks caused by changes in pairing (described previously) are treated as permanent, and the β-strands on either side are kept in separate β-sheets. Breaks caused by geometric evaluation of the local region are treated as potential breaks. A geometric break is ignored unless it affects all paired residues on either side of it. Thus, geometric breaks are used only if multiple β-strands need to be broken to split the β-sheet. Keeping a rigid criterion for the use of geometric breaks for individual β-strands ensures flexibility for the entire β-sheet, as larger β-sheets tend to show a gradual bending. A more drastic bending shows up as a sequence of geometric abnormalities, thus allowing proper use of the potential geometric breaks detected previously.

Assignment of β-strands to β-sheets is for ease of motif searches only. Our program does not define β-sheets for the purpose of domain definition. As such, ambiguity regarding whether two β-sheets should be linked or separate might arise in cases where there is a gradual bending of the sheet or where two or more β-strands link them at the edges. It is also possible for two different β-sheets to be linked together if a few β-strands in each sheet are distorted and are in close proximity to each other. In the majority of cases, however, our program defines β-sheet boundaries correctly.

### Robustness of secondary structure assignment towards coordinate errors

Robustness towards coordinate errors was estimated by checking consistency of definition using randomly shifted PDB [20] coordinates. Gaussian random numbers were used to shift coordinates of the 100 files in the culled PDB set. Every residue (all atoms together) was individually shifted in all the three axes. 100 randomly shifted coordinate files were generated for every PDB file, starting each time with the original coordinates. 10 sets of 10,000 files each were generated with average RMSD from 0.2Å to 2.0Å in steps of 0.2Å. Secondary structure definition produced by DSSP [8], P-SEA [17] and our algorithm for each of these 1,00,000 files was compared with the definition produced by the same program from the unaltered coordinates. Mean percentage of correct definition over all residues, assuming definition from the unaltered definition to be fully correct, and standard error was calculated for each set of 10,000 files for each program (fig. 2a). Mean percentage of total residues reported as helices and β-strands for each of these 10 sets were also noted (fig. 2b).

## Authors' contributions

IM designed and implemented the algorithms, tested program performance, analyzed the results, and drafted the manuscript. SSK contributed to algorithm development and provided expert judgment of program output. NVG conceived of the study and participated in its design and coordination. All authors read and approved the final manuscript.
